# A novel method to measure steroid hormone concentrations in walrus bone from archaeological, historical, and modern time periods using liquid chromatography/tandem mass spectrometry

**DOI:** 10.1002/rcm.8272

**Published:** 2018-11-06

**Authors:** Patrick Charapata, Lara Horstmann, Amber Jannasch, Nicole Misarti

**Affiliations:** ^1^ College of Fisheries and Ocean Sciences University of Alaska Fairbanks PO Box 757220 Fairbanks AK 99775 USA; ^2^ Bindley Bioscience Center Purdue University 1203 W State St. West Lafayette IN 47906 USA; ^3^ Water and Environmental Research Center University of Alaska Fairbanks PO Box 755910 Fairbanks AK 99775 USA; ^4^ Department of Biology Baylor University One Bear Place Waco TX 76706 USA

## Abstract

**Rationale:**

A liquid chromatography/tandem mass spectrometry (LC/MS/MS) method was validated and utilized to measure and analyze four steroid hormones related to stress and reproduction in individual samples from a novel tissue, Pacific walrus (Odobenus rosmarus divergens, herein walrus) bone. This method determines steroid hormone concentrations in the remote walrus population over millennia from archaeological (>200 bp), historical (200–20 bp), and modern (2014–2016) time periods.

**Methods:**

Lipids were extracted from walrus bone collected from these periods using methanol before LC/MS/MS analysis. Isotopically labeled internal standards for each target hormone were added to every sample. Analytical and physiological validations were performed. Additionally, a tissue comparison was done among paired walrus bone, serum, and blubber samples. A rapid resolution liquid chromatography system coupled to a QqQ mass spectrometer was used to analyze all samples after derivatization for progesterone, testosterone, cortisol, and estradiol concentrations. Multiple reaction monitoring was used for MS analysis and data were acquired in positive electrospray ionization mode.

**Results:**

Progesterone, testosterone, cortisol, and estradiol were linear along their respective standard calibration curves based on their R^2^ values (all > 0.99). Accuracy ranged from 93–111% for all hormones. The recovery of extraction, recovery of hormones without matrix effect, was 92–101%. The overall process efficiency of our method for measuring hormones in walrus bone was 93–112%. Progesterone and testosterone concentrations were not affected by reproductive status among adult females and males, respectively. However, estradiol was different among pregnant and non‐pregnant adult females. Overall, steroid hormones reflect a long‐term reservoir in cortical bone. This method was also successfully applied to walrus bone as old as 3585 bp.

**Conclusions:**

LC/MS/MS analysis of bone tissue (0.2–0.3 g) provides stress and reproductive data from elusive walruses that were alive thousands of years ago. Based on physiological validations, tissue comparison, and published literature, steroid hormone concentrations measured in walrus cortical bone could represent an accumulated average around a 10–20‐year time span. By investigating how stress and reproductive physiology may have changed over the past ~3000 years based on bone steroid hormone concentrations, this method will help answer how physiologically resilient walruses are to climate change in the Arctic.

## 1 INTRODUCTION

Lipophilic steroid hormones, including cortisol, estradiol, progesterone, and testosterone, can provide important physiological information in marine mammals. Estradiol, progesterone, and testosterone are reproductive hormones and have been used to determine marine mammal reproductive status, including pregnancy in cetaceans,^1^, ^2^, ^3^, ^4^ pinnipeds,^5^, ^6^ and Pacific walruses (Odobenus rosmarus divergens, hereafter walrus).^7^ Cortisol is produced in response to stress and naturally increases during times of high energy use including reproduction, molting, and mobilization of lipid stores in pinnipeds.^5^, ^6^, ^8^, ^9^, ^10^ However, when cortisol concentrations are consistently high, this is an indicator of a chronic stressor.^11^ Pinnipeds are more susceptible to disease and may have poorer body condition when experiencing chronic stress compared to animals that have not been exposed to a chronic stressor and experience continuously elevated cortisol concentrations.^12^, ^13^ Clearly, steroid hormone studies of marine mammals provide relevant physiological data for current and future population health assessments.^14^

Bone tissue contains lipids which are sequestered over the lifespan of an animal and do not significantly degrade after death, which means they can be detected in the bone after being buried for thousands of years.^15^, ^16^ Testosterone and estrogens, including estradiol, have been extracted and used to assign sex to human bones as old as 6961 calendar years before present (bp),^17^ and testosterone and estradiol have been extracted and analyzed from rat (*Rattus* spp.) bone.^18^ Bone has a slow turnover rate (3% cortical bone/year),^19^ therefore hormone concentrations from bone are expected to represent a long‐term accumulated average for an individual. This is beneficial when monitoring long‐term physiological changes in a population, because bone hormone concentrations are not likely to be skewed by acute stressors or reproductive events like serum and blubber.^2^, ^3^, ^8^, ^20^, ^21^ While steroid hormones have not yet been extracted from marine mammal bone from archaeological (> 200 bp), historical (200–20 bp), or modern (2014–2016 CE) time periods, lipids, including cholesterol, have been obtained from ancient whale bone (75000 bp),^15^ and steroid hormones have been extracted from rat bone and measured using enzyme immunoassays (EIAs).^18^

Steroid hormones have been extracted from numerous matrices and measured with various immunoassay kits including enzyme linked immunoassays (ELISAs), radioimmunoassays (RIAs), and EIAs. Assays have been used to measure steroid hormones in marine mammal feces,^1^, ^22^ blubber,^6^ serum, urine,^21^ saliva,^7^, ^23^ baleen,^4^, ^24^ earwax,^25^ and whale blow.^26^ Immunoassay techniques are beneficial when sample mass is abundant, and they generally lower the cost of analysis.^27^ However, immunoassays require relatively large sample masses and multiple assays for multi‐hormone analyses, which increases required lab time.^28^ In addition, cross‐reactivity with target steroid hormone metabolites can lead to inflated hormone concentrations.^29^, ^30^ Furthermore, due to complicated logistics of collecting tissue samples from free‐ranging marine mammals and animal care standards for managed populations, marine mammal biopsies, blow samples, fecal samples, etc., once obtained, are relatively small and are slated for multiple different analyses, (e.g., contaminants, fatty acids, disease).^31^, ^32^ Thus, researchers need to efficiently analyze tissue samples and have been transitioning from using immunoassays to more sophisticated analyses, like liquid chromatography/tandem mass spectrometry (LC/MS/MS).^28^, ^30^

LC/MS/MS analysis allows for greater utility of samples collected from rarely encountered species, such as marine mammals. For example, eight different hormones have been analyzed in a single 0.40 g blubber sample using LC/MS/MS^28^ compared with a single hormone being measured in 0.15 g using ELISAs.^33^ LC/MS/MS measures the amount of the actual target analyte, resulting in low cross‐reactivity of metabolites and greater accuracy of actual steroid hormone concentrations in samples.^34^ Recently, a variety of LC/MS/MS methods have been developed to measure multiple hormones in a single sample of marine mammal blubber,^28^, ^30^, ^34^ whale blow,^35^ and serum.^34^, ^36^ Serum represents circulating hormone concentrations (short‐term), while blubber represents approximately weekly to monthly hormone concentrations.^6^, ^21^, ^36^, ^37^, ^38^ Here, we developed a LC/MS/MS method for measuring steroid hormones in walrus bone collected during archaeological (> 200 bp), historical (200–20 bp), and modern (2014–2016 CE) time periods in an effort to monitor long‐term changes in walrus physiology.

In this study, steroid hormone concentrations from archaeological, historical, and modern walrus bone were measured utilizing positive electrospray ionization (ESI), derivatizations of steroid hormones, and multiple reaction monitoring during LC/MS/MS analysis. Our objectives were to: (1) validate a method of lipid extraction and LC/MS/MS for analyzing steroid hormone concentrations from walrus bone, (2) determine physiological validations of bone steroid hormones in walrus bone based on known reproductive status and a tissue comparison among paired serum, blubber, and bone samples, and (3) apply this LC/MS/MS method to measure steroid hormones in walrus bone from the three time periods. This study provides the first results of steroid hormone concentrations extracted from marine mammal bone, including archaeological walrus bone as old as 3585 bp. This LC/MS/MS method measures four steroid hormones in a single, small‐mass walrus bone sample, resulting in efficient collection of physiological data from rare archaeological and museum specimens. This method potentially provides a new long‐term tool for monitoring cortisol and reproductive hormone concentrations of walruses and other marine and terrestrial mammals.

## 2 EXPERIMENTAL

### 2.1 Chemicals and reagents

Lipid extraction of powdered bone was performed by using 100% HPLC grade methanol from VWR BDH® Chemicals (Radnor, PA, USA). Isotopically labeled internal standards, d_4_‐cortisol, ^13^C_3_‐testosterone, ^2^H_9_‐progesterone, and ^2^H_5_‐estradiol, were obtained from Sigma‐Aldrich (St Louis, MO, USA). Non‐isotopically labeled hormones used to create calibration curves were also acquired: hydrocortisone, β‐estradiol, and testosterone from Sigma‐Aldrich and progesterone from Calibiochem (San Diego, CA, USA). HPLC grade methanol for LC/MS/MS analysis performed at Bindeley Science Center at Purdue University was supplied by Fisher Chemicals (Fair Lawn, NJ, USA). Dansyl chloride and acetone for the dansyl chloride solution for the derivation of samples were purchased from Sigma‐Aldrich. Sodium carbonate added to samples with dansyl chloride solution was procured from Sigma‐Aldrich. Formic acid and acetonitrile used as buffer solutions during LC/MS/MS analysis were from Sigma‐Aldrich and Fisher Chemicals, respectively. Keto derivatives were prepared using the Amplifex keto reagent (AB Sciex, Framingham, MA, USA).

### 2.2 Sample collection and permits

#### 2.2.1 Sample collection

Archaeological (> 200 bp), historical (200–20 bp) and modern (2014–2016 CE) walrus cortical bone samples were used for the extraction, validation, and measurement of steroid hormones (see overall sample sizes for bone in Table 1). Archaeological walrus bones derived from various sites throughout the Alaskan and Russian walrus habitat (Appendix 1) were obtained through the University of Alaska Museum (UAM) Archaeological Collection and Dr. A. Jensen at Ukpeaġvik Iñupiat Corporation (UIC) in Utqiaġvik, Alaska (Appendix 1). Historical samples were acquired from the UAM Mammal Collection and the Smithsonian Institution National Museum of Natural History (Appendices 1 and 2). Modern samples were collected from Native subsistence harvests on St Lawrence Island, AK, USA, through an agreement with Native hunters, the Eskimo Walrus Commission, the Alaska Department of Fish and Game (ADF&G), and the U.S. Fish and Wildlife Service (USFWS) during April and May 2014–2016 (Appendices 1 and 2). Hunters recorded sex, age class, and reproductive information for harvested females (i.e., presence of a fetus, calf, yearling, and/or lactating). Bone samples were transferred to the University of Alaska Fairbanks (UAF) for sample analysis under a Letter of Authorization from the U.S. Fish and Wildlife Service to Dr. L. Horstmann. Additional modern samples were opportunistically collected (Appendix 1) in partnership with the North Slope Borough Department of Wildlife Management and Native subsistence hunters from Utqiaġvik.

**Table 1:** Total sample sizes (*n*) of walrus bones collected for analyses during archaeological, historical, and modern time periods. Further, sample sizes are categorized into age class (adult, subadult, and unknown) and sex (female, male, and unknown) for each time period. “‐“ indicates no samples were collected for that category. See other tables for specific sample sizes for physiological validation (Table 3), time period (Table 6), and tissue comparison analyses (Tables 7 and 8)

	Adult			Subadult			Unknown			
	Archaeological	Historical	Modern	Archaeological	Historical	Modern	Archaeological	Historical	Modern	Totals
**Female**	‐	24	19	‐	17	1	‐	‐	1	62
**Male**	‐	4	29	‐	2	9	‐	‐	3	47
**Unknown**	10	‐	‐	‐	‐	‐	‐	‐	‐	10
Totals	10	28	48	‐	19	10	‐	‐	4	119

Paired blubber and serum samples were collected along with the modern bone samples from April and May of 2014–2015 for tissue comparison analysis (Appendix 3). Full thickness blubber with skin and muscle attached as well as blood were collected from an area of the walrus body that the hunters deemed adequate for collection, generally sternal blubber. Bone from these same animals was generally a metatarsal or metacarpal bone. 25 mL of blood was collected in Falcon tubes containing anti‐coagulating glass beads (MS4491, Market Lab Inc., Caledonia, MI, USA). Blubber, blood, and bone samples were kept at ambient temperature (~ −12°C) until hunters returned to town. Blood was spun in a centrifuge within 8 hrs of collection with serum collected and frozen at −20°C. Samples were shipped frozen to the Marine Mammal Laboratory at UAF and immediately transferred to −80°C until steroid hormone analysis.

### 2.3 Steroid hormone extraction

#### 2.3.1 Bone samples

All archaeological, historical, and modern walrus bones were extracted for steroid hormone analysis following the procedure outlined below. Sections of bone were polished with a Dremel® 3000 drill with a sand drum attachment to remove outside contaminants exposing clean areas of cortical bone. Approximately 1.5 g of cortical bone was removed using the Dremel drill with a diamond blade attachment. Pieces of bone were pulverized into powder using a Wig‐L‐Bug®, and 0.2–0.3 g of powdered bone were transferred to 2.8 mL ceramic bead homogenizer cryovials. Sample weights were recorded to the nearest 0.0001 g. Samples were homogenized, dry, on a Disruptor Genie® (Scientific Industries, Bohemia, NY, USA) for 1 min. Samples were spiked with 100 ng of isotopically labeled internal standards (Sigma‐Aldrich) (ISTD), d_4_‐cortisol, ^13^C_3_‐testosterone, ^2^H_9_‐progesterone, and ^2^H_5_‐estradiol, for accurate hormone detection and validation during LC/MS/MS analysis.^27^, ^29^, ^35^, ^39^ Lipid extraction of the powdered bone was performed by adding 1.460 mL of methanol (BDH).^4^, ^40^, ^41^ Samples were homogenized for 3 min on a Disruptor Genie® (Scientific Industries) and set on a rocking platform (VWR®; model 100) for 24 h. Samples were then centrifuged (Microfuge® 18 centrifuge, Beckman Coulter, Brea, CA, USA) at 12000 RPMs (13000 *g*) for 20 min. Supernatant from each sample was pipetted into glass vials and dried using nitrogen gas (N‐EVAP™112, Organomation Associates, Inc., Berlin, MA, USA) leaving only lipids. Samples were then stored in a − 80°C freezer until analysis.

#### 2.3.2 Blubber samples

The oxidized outer layer of walrus blubber from each full thickness slab was removed with sterilized individual razor blades exposing fresh blubber tissue. Two separate vertical strips of full thickness blubber weighing between 0.2–0.3 g were removed starting from below the skin and ending above the muscle and transferred to separate 2.8 mL ceramic bead homogenizer cryovials. Samples were homogenized, internal standards added, and lipids extracted with methanol as described above, except samples were vortexed for 8 min after methanol and internal standards had been added to samples. Sample extracts were stored in a − 80°C freezer until being shipped for LC/MS/MS analysis. Blubber samples were run in duplicate with the average concentration (ng/g blubber) used for analysis. There were a total of 32 blubber samples with 5 females and 27 males.

#### 2.3.3 Serum samples

Serum was thawed and mixed before steroid hormone extractions. For each serum sample, 375 μL of serum was added to 2.8 mL ceramic bead cryovials. Samples were spiked with internal standards and extracted using methanol as described above for bone samples. Sample extracts were stored in a − 80°C freezer until being shipped for LC/MS/MS analysis. Serum samples were run in duplicate with the average concentration (ng/mL serum) used for analysis. There were a total of 22 serum samples with 6 females and 14 males.

### 2.4 Steroid hormone concentrations among different bone elements

To ensure that steroid hormone concentrations do not differ between walrus skeletal elements, we performed a pilot study on skull and mandible bone sampled from the same individual walruses (*n* = 7). All steroid hormone concentrations were similar between skulls and mandibles from the same individual (paired t‐tests; cortisol *P* = 0.32, estradiol *P* = 0.08, progesterone *P* = 0.20, and testosterone *P* = 0.11, n = 7 pairs**)**. These data agree with Yarrow et al,^18^ where testosterone measured in tibias and femurs of rats were similar. This pilot study confirmed that different walrus skeletal elements used here result in comparable steroid hormone concentrations.

### 2.5 Percent lipid correction factor

Walrus bones from different archaeological, historical, and modern time periods potentially have different lipid compositions, as lipid in cortical bone is already low,^19^, ^42^ and taphonomic processes could affect the lipid composition of archaeological bones buried for thousands of years.^15^, ^43^ In addition, there has been evidence of degradation of progesterone in cetacean blubber,^3^ which contains lipid‐associated hormones similar to bone. Therefore, steroid hormones most likely degrade over millennia in bone and need to be corrected for lipid degradation and leeching to compare steroid hormone concentrations across thousands of years. A mean percent lipid correction factor was used to correct potential lipid composition differences among time periods. Bones (*n* = 12, 10, and 12, for archaeological, historical, and modern bone, respectively) from each sample time period were lipid extracted using a modified (2:1 chloroform/methanol) Soxhlet procedure after Schlechtriem et al.^44^ A one‐way analysis of variance (ANOVA) followed by Tukey's pairwise comparison determined that mean percent lipid of modern bone (4.83 ± 1.78%) was significantly higher than archaeological (2.71 ± 1.96%) and historical (1.98 ± 1.52%) walrus bone (*P* = 0.02, 0.002, respectively). Therefore, steroid hormone concentrations from all samples were corrected by mean percent lipid weight based on their sample time period and are reported as ng/g lipid. Hormone concentrations are also reported as the more traditional ng/g bone for reference and tissue comparison purposes.

### 2.6 LC/MS/MS conditions and analysis of steroid hormones

Prior to analysis, each sample was reconstituted in 200 μL of methanol, split into two equal aliquots and dried again using an Eppendorf‐Vacufuge rotary evaporating device. The first aliquot of each extract was derivatized with dansyl chloride according to Zhang et al^45^ just prior to LC/MS/MS analysis. To each sample, 20 μL of 10mM Na_2_CO_3_ and 50 μL of freshly prepared dansyl chloride solution (3 mg/mL acetone) were added. The samples were heated at 60°C for 10 min, transferred to autosampler vials, and immediately analyzed. The second aliquot of each extract was derivatized with the AB Sciex keto derivatization kit (AB Sciex) just prior to LC/MS/MS analysis, and 50 μL of reagent was added. The reaction time was 60 min at room temperature. Finally, the samples were transferred to autosampler vials and immediately analyzed.

An Agilent 1200 rapid resolution liquid chromatography (LC) system coupled to an Agilent 6460 series QqQ mass spectrometer (MS) was used to analyze all samples after derivatization at the Bindeley Bioscience Center at Purdue University, IN, USA. For the dansyl chloride derivatives, the following conditions were used. A Waters Xbridge C18 column (2.1 mm × 100 mm, 3 μm) was used for LC separation. The buffers were (A) water +0.1% formic acid and (B) acetonitrile +0.1% formic acid. The linear LC gradient was as follows: time 0 min, 90% A; time 5 min, 0% A; time 15.5 min, 90% A; time 18 min, 90% A. The flow rates of buffers A and B were 0.3 mL/min. Multiple reaction monitoring was used for MS analysis. The data were acquired in positive electrospray ionization (ESI) mode by monitoring the following transitions: estradiol (dansyl Cl), *m/z* (atomic mass units) 506.1→171 (30 V), *m/z* 155.8 (40 V); ^2^H_5_‐estradiol (dansyl Cl), *m/z* 511.1→171 (30 V), *m/z* 155.8 (40 V); estriol (dansyl Cl), *m/z* 522→171 (30 V), 155.8 (40 V). This method can also be used to monitor progesterone in its unlabeled form by following the transition: *m/z* 315.2→109 (15 V), 97 (15 V); ^2^H_9_‐progesterone, *m/z* 324.2→113 (15 V), 100 (15 V) if necessary. The ESI interface had a nitrogen gas temperature of 325°C, nitrogen gas flow rate of 8 L/min, nebulizer pressure of 45 psi, sheath gas temperature of 250°C, sheath gas flow rate of 7 L/min, capillary voltage of 3500 V, and nozzle voltage of 1500 V.

For the keto derivatives, the following conditions were used for LC/MS/MS analysis. An Agilent Zorbax 80 Å Extend‐C18 column (4.6 mm × 150 mm, 5 μm) was used with buffers A (water +0.1% formic acid) and B (acetonitrile +0.1% formic acid). The linear LC gradient was as follows: time 0 min, 90% A; time 10 min, 0% A; time 12 min, 90% A; time 15 min, and 90% A. The flow rates of buffers A and B were 0.8 mL/min. Multiple reaction monitoring was used for MS analysis. The data were acquired in positive ESI mode by monitoring the following transitions: testosterone, *m/z* 403.1→344.1 (20 V), 164 (40 V); ^13^C_3_‐testosterone *m/z* 406.1→347.1 (20 V), 167 (40 V); cortisol *m/z* 477.1→418.3 (15 V), 388.2 (35 V); d_4_‐cortisol *m/z* 481.1→422.3 (15 V), 392.3 (35 V); progesterone *m/z* 429.1→370 (20 V), 126 (30 V); ^2^H_9_‐progesterone *m/z* 438.1→379 (20 V), 132 (30 V). The jet stream ESI interface had a nitrogen gas temperature of 325°C, nitrogen gas flow rate of 8 L/min, nebulizer pressure of 45 psi, sheath gas temperature of 250°C, sheath gas flow rate of 7 L/min, capillary voltage of 4000 V, and nozzle voltage of 1000 V.

Samples with hormone concentrations below the detection limit for LC/MS/MS analysis (< 2.0 ng/g), were included in statistical analysis by assigning one‐half the detection limit concentrations for each hormone with a non‐detectable signal.^46^, ^47^ Extraction efficiencies were determined by comparing known volumes of added internal standards of each hormone that had been through the extraction process (i.e., blank samples that went through the steroid hormone extraction method with only added internal standards and methanol, *n* = 8, “Blank‐Extraction”), with samples with internal standards and no extraction (i.e., added internal standard to vial and dried using nitrogen gas, *n* = 5, “Blank‐Dried Internal Standards”).

### 2.7 Preparation of standards and stock solutions

Stock vials of isotopically labeled internal standards, d_4_‐cortisol, ^13^C_3_‐testosterone, ^2^H_9_‐progesterone, and ^2^H_5_‐estradiol, were diluted to 10 ng/μL with methanol in separate 10 mL glass scintillation vials. Glass vials were then wrapped in aluminum foil and stored at −8°C. Non‐labeled steroid hormone standards (hydrocortisone, β‐estradiol, testosterone, and progesterone) used for creating calibration curves were diluted with methanol to both 10 ng/μL and 0.05 ng/μL per steroid hormone. Non‐labeled steroid hormone standards were kept in amber 1 L bottles and stored at −8°C. All standards and stock solutions were brought to room temperature before analysis.

### 2.8 Analytical validation of steroid hormones in bone

Steroid hormones extracted from walrus bone were validated for linearity, accuracy, matrix effects, precision, and extraction efficiencies according to Zhang et al^45^ and Caban et al.^48^ Briefly, bone powder was pooled from each time period based on availability of excess bone powder from samples (around 4.2 g of bone powder/time period). Standard curves were created based on minimum detection limits of the LC/MS/MS instrument (< 0.5 ng) to twice the maximum steroid hormone concentrations measured in walrus samples from this study. Progesterone had the highest concentrations measured, and thus had eight standards along its calibration curve (0.5, 50, 100, 200, 400, 800, 1600, and 3200 ng). Testosterone and cortisol had five standards making up the standard curve (0.5, 75, 125, 250, and 500 ng). Estradiol had five standards along its calibration curve (5, 25, 50, 100, and 200 ng). There were replicates (*n* = 5) for all concentrations along the calibration curves for each hormone. A blank standard calibration curve was created for comparison with pooled bone samples that were spiked with non‐isotopically labeled steroid hormones along each of the four calibration curves for each hormone. Additional (*n* = 5) pooled bone powder samples were spiked post‐extraction with a middle standard concentration of each non‐isotopically labeled hormone (400, 125, 125, and 50 ng for progesterone, testosterone, cortisol, and estradiol, respectively). All samples went through the same extraction method as described above, including the addition of 100 ng of isotopically labeled internal standard for each hormone.

**Figure 1:**
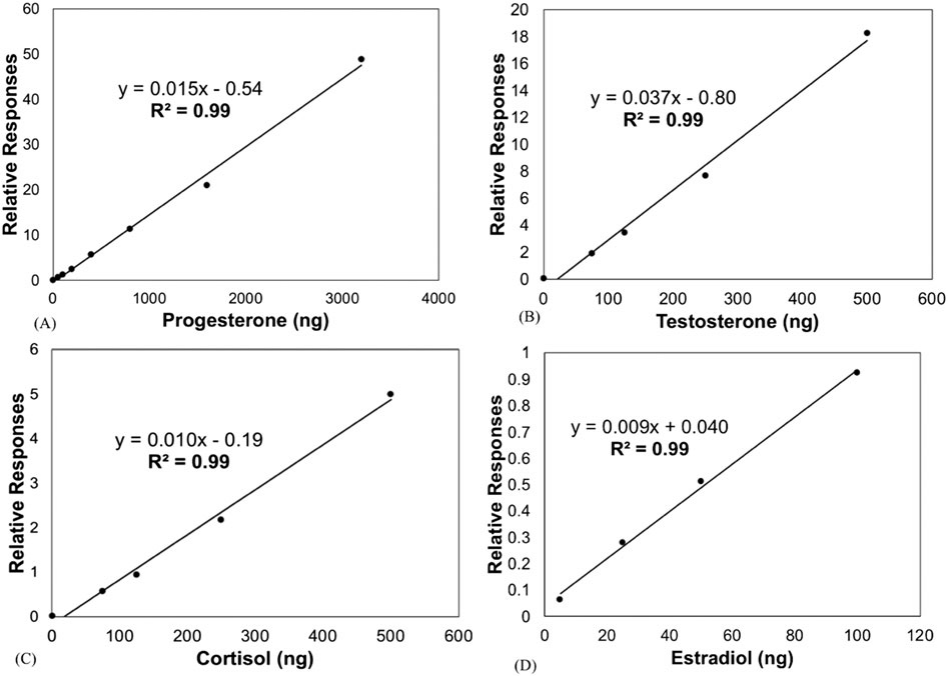
Linear responses of progesterone (A), testosterone (B), cortisol (C), and estradiol (D) based on mean (*n* = 5, for each standard) relative responses (ratio of peak area ratios of analyte divided by the peak area of isotopically labeled internal standard) of each hormone from walrus bone spiked with each hormone along a standard calibration curve (as stated in section 2.8) according to Zhang et al.^45^ Linear regression equations along with R^2^ values are provided for each steroid hormone

Linearity was determined by plotting the mean relative response ratios (*n* = 5) from bone powder spiked with concentrations of each hormone along their respective standard calibration curves (Figure 1).^45^ The mean relative response ratio is the peak area ratio of the analyte divided by the peak area of isotopically labeled internal standard. Accuracy was determined by using the equation:

(1) $$\text{Accuracy}=\left(\mathrm{EC}/\mathrm{MAC}\right)*100$$

Where expected concentration (EC) is divided by mean actual concentration measured in spiked bone tissue (MAC) and then multiplied by 100 (Table 2).^45^ Steroid hormone concentrations in marine mammal serum and blubber have been previously validated using LC/MS/MS methods.^28^, ^30^, ^34^

**Table 2:** Mean concentrations, coefficient of variation (CV), and mean accuracy of measured steroid hormones in walrus bone tissue spiked with each hormone's respective concentrations along a standard calibration curve (i.e., “Concentration (ng)”). Respective concentrations for each hormone have *n* = 5 replicates. Accuracy was determined following Zhang et al^45^

Hormone	Concentration (ng)	Mean concentration (ng)	CV (%)	Mean accuracy (%)
Progesterone	3200	3206.04	9.33	100.19
	1600	1535.04	0.98	95.94
	800	869.73	6.50	108.72
	400	445.19	6.69	111.30
	200	199.90	6.11	99.95
	100	102.58	5.70	102.58
	50	50.13	7.67	100.25
	0.5	0.53	3.39	105.54
Testosterone	500	499.76	1.84	99.95
	250	251.30	4.17	100.52
	125	124.73	2.19	99.78
	75	71.92	1.61	95.92
	0.5	0.54	3.15	107.07
Cortisol	500	498.09	4.03	99.62
	250	255.25	2.99	102.10
	125	125.19	1.10	100.15
	75	69.65	3.14	92.87
	0.5	0.54	8.30	107.50
Estradiol	200	200.90	2.64	100.45
	100	100.22	3.54	100.22
	50	48.99	7.96	97.98
	25	25.24	3.64	100.97
	5	5.40	8.56	107.99

### 2.9 Physiological validation of steroid hormones in bone

Physiological validations of steroid hormones were carried out using walrus bones of known sex and reproductive status obtained from museum archives and Native subsistence harvests. Using known females of different reproductive status, progesterone, estradiol, and cortisol were compared to determine if these hormones were higher in pregnant animals,^4^, ^7^, ^22^, ^49^ and varied among three physiological states: subadult females, non‐pregnant females, and pregnant females (Tables 3A and 3B). Any other additional reproductive status information was noted for analysis (lactating, calf present, etc., Tables 3A and 3B). Females were classified as subadult based on provenience data from museum records, hunter observations, and tooth age estimates,^50^ where sexually immature status is assigned to walruses approximately 1–9 years old^51^ (Appendix 2). Classification as non‐pregnant adult female was based on tooth age estimates, no fetus being present based on hunter observations, and/or museum records (Appendix 2). Animals classified as pregnant included only females with a fetus based on hunter observations and/or museum records. In addition, testosterone concentrations were compared from known subadult males (*n* = 11, 3–14 years)^51^ with known adult males (Table 3A, *n* = 28, 15–28 years).^51^ Classification groups for males were based on tooth age estimates only. These physiological validations lend evidence to better estimate reservoir time of steroid hormones in cortical bone. All bone samples used for physiological validations are listed with provenience data in Appendix 2.

**Table 3:** Median ± 1 SD, ranges (minimum – maximum), *P*‐values (Kruskal‐Wallis ANOVAs, bold if significant) of steroid hormones measured in walrus bone from females of different ages and differing reproductive status along with subadult males (3–14 years, *n* = 11)^51^ and adult males (15–28 years, *n* = 28).^51^ (A) Pregnant females were defined as females with a fetus. Different reproductive information is provided for pregnant females (lactating and/or offspring were present [Y/N]). “*” includes one female that was lactating with a yearling. “‐” represents no available data. *P*‐values for comparison among subadult and adult females of different reproductive status are presented. Females were classified as subadult based on provenience data provided by museum records, hunter observations, and tooth age estimates^50^ (sexually immature from approximately 1–9 years old).^51^ Non‐pregnant adult females were classified as adult based on tooth ages (i.e., > 9 years) and no fetus being present based on hunter observations. There were similar concentrations among adult females irrespective of pregnancy and reproductive status, except for estradiol (A). *P*‐values (Kruskal‐Wallis ANOVAs, bold if significant) for differences in estradiol concentrations among females of different age class and reproductive status (B). Abbreviations correspond to category of female in (A): not pregnant (NP), pregnant (YP), calf present (C), yearling present (Y), no calf or yearling present (NCY), lactating (L), unknown with respect to pregnancy or young present (UNK), and subadult (SA). For example, NP_C_L* are adult non‐pregnant (NP) females with a calf present (C) and are lactating (L). “*” represents the one adult non‐pregnant female that was lactating and had a yearling present

(A)									
Sex	Age class	Pregnancy	Lactating	Calf or yearling?	Sample size (n)	Hormone	Median ± SD mean (ng/g lipid)	Range (min ‐ max) (ng/g lipid)	*P*‐value (compared with subadults)
Female	Adult	No	Yes	Calf*	8	Progesterone	149.48 ± 500.05 308.73	19.11–1526.26	**<0.001**
	Adult	No	No	Both	1		19.16	‐	0.17
	Adult	No	No	None	4		211.80 ± 219.88 246.35	20.68–541.12	**0.02**
	Adult	No	Unknown	Unknown	16		363.43 ± 486.60 560.94	128.07–1971.63	**<0.001**
	Adult	Yes	Y/N	Y/N	8		356.13 ± 1190.60 979.21	101.16–3414.99	**0.01**
	Subadult	Unknown	Unknown	Unknown	18		7381.86 ± 7743.49 8841.24	448.79–30329.86	‐
Female	Adult	No	Yes	Calf*	8	Testosterone	141.58 ± 212.47 212.75	80.06–722.84	**<0.001**
	Adult	No	No	Both	1		62.49	‐	0.12
	Adult	No	No	None	4		185.05 ± 100.40 186.19	67.20–307.48	**0.004**
	Adult	No	Unknown	Unknown	16		288.89 ± 114.50 289.42	127.90–543.13	**<0.001**
	Adult	Yes	N/A	N/A	8		323.78 ± 160.91 320.46	73.20–541.95	**<0.001**
	Subadult	Unknown	Unknown	Unknown	18		2497.74 ± 2278.47 2660.09	178.61–7621.84	‐
Female	Adult	No	Yes	Calf*	8	Cortisol	44.40 ± 112.85 77.82	11.86–349.71	**0.008**
	Adult	No	No	Both	1		6.43	‐	0.12
	Adult	No	No	None	4		76.81 ± 64.45 80.83	16.27–153.46	0.12
	Adult	No	Unknown	Unknown	16		33.70 ± 14.35 33.54	13.61–63.12	**<0.001**
	Adult	Yes	N/A	N/A	8		29.45 ± 25.48 38.87	15.67–88.70	**<0.001**
	Subadult	Unknown	Unknown	Unknown	18		158.51 ± 2440.12 968.19	32.10–10412.57	‐
Female	Adult	No	Yes	Calf*	8	Estradiol	2373.87 ± 1250.05 1531.60	19.12–2483.80	See Table 3B
	Adult	No	No	Both	1		19.16	‐	See Table 3B
	Adult	No	No	None	4		18.62 ± 1.39 18.83	17.41–20.68	See Table 3B
	Adult	No	Unknown	Unknown	16		48.32 ± 20.90 53.94	21.93–104.41	See Table 3B
	Adult	Yes	N/A	N/A	8		131.89 ± 1097.55 695.63	18.09–2567.74	See Table 3B
	Subadult	Unknown	Unknown	Unknown	18		6523.68 ± 2959.51 5982.63	18.36–9460.71	See Table 3B
Male	Adult	NA	NA	NA	28	Testosterone	317.21 ± 141.05 268.37	33.96–1333.68	0.34
	Subadult	NA	NA	NA	11		246.88 ± 178.49 270.52	34.94–674.84	

(B)						
Estradiol						
	NP_C_L*	NP_CY	NP_NCY	NP_UNK	SA	YP
NP_C_L*	‐	‐	‐	‐	‐	‐
**NP_CY**	0.19	‐	‐	‐	‐	‐
**NP_NCY**	**0.02**	0.72	‐	‐	‐	‐
**NP_UNK**	0.10	0.13	**<0.001**	‐	‐	‐
**SA**	**<0.001**	0.17	**<0.001**	**<0.001**	‐	‐
**YP**	0.39	0.33	**0.03**	**0.01**	**<0.001**	‐

Serum has been associated with circulating concentrations of steroid hormones, while blubber is a longer‐term reservoir accumulating large pulses of steroid hormones originating from serum during pregnancy and molting events, and then equalizing with serum concentrations thereafter.^10^, ^21^ Cortical bone is a reservoir for steroid hormones, but residency time of hormones in cortical bone is unknown.^18^ A tissue comparison among paired bone, blubber, and serum samples was performed to help determine how bone concentrations compare with tissues of well‐studied steroid hormone residency times, complementing the bone physiological validations (discussed above). All paired tissue samples used in the tissue comparison are listed in Appendix 3. Bone concentrations were reported as ng/g bone for accurate comparisons with serum (ng/mL serum) and blubber (ng/g blubber).

### 2.10 Statistical analysis

Steroid hormone concentrations in walrus bone were not normally distributed; therefore, non‐parametric Kruskal‐Wallis ANOVAs were used to determine significant differences in hormone concentrations among known subadult and adult females of different reproductive statuses and between adult and subadult males to perform physiological validations (as described above). Kruskal‐Wallis ANOVAs analyze differences in median values that are robust to outliers. All physiological validation data are reported as median ± 1 standard deviation (SD), with mean values reported for reference in ng/g lipid (Tables 3A and 3B). The samples used for the physiological validations are listed in Appendix 2.

The data used for tissue comparison analysis were log transformed to normalize distribution of steroid hormone concentrations in bone, blubber, and serum. The samples used for the tissue comparison are listed in Appendix 3. Three factorial ANOVA tests were used to test for differences in mean concentrations of steroid hormones among all tissues, between sexes, and the interaction of sex and tissue. If the differences among hormone concentrations were statistically significant among tissues, between sexes, and/or had a significant interaction of tissue and sex, a Tukey post hoc test was used to elucidate specific differences among the factors (“tissue”, “sex”, “sex*tissue”) for each hormone. The majority of animals were classified as adult walruses (*n* = 25 adults, *n* = 3 subadults, and *n* = 5 unknown); therefore, sample size was too small to perform statistical analysis among age classes (Appendix 3). Tissue comparison data were reported as ng/g (blubber and bone) and ng/mL (serum). All statistics were performed in PAST (V3.14).^52^ An alpha of 0.05 was used for all analyses. Any samples with concentrations below detectable limits (< 2.0 ng/g) were included in all analyses by assigning one‐half the detectable concentration.^46^, ^53^ Linear regressions were run among hormones measured in paired tissues to determine if any correlations existed among bone, blubber, and serum hormone concentrations. The only significant model was male bone and serum progesterone concentrations (linear regression, R^2^ = 0.51, *P* < 0.001; other data not shown *P* > 0.05). Additional subadults for the paired tissue comparison would have potentially increased the range in hormone concentrations, improving the linear correlation analyses.

## 3 RESULTS AND DISCUSSION

### 3.1 Analytical validation of steroid hormones in bone

Steroid hormones were linear along their respective standard calibration curves based on their R^2^ values (all R^2^ > 0.99, Figure 1).^45^ Estradiol was linear from 5–100 ng, but not up to 200 ng. While this has no bearing on our results (maximum raw estradiol value from all walrus bone samples was 62.93 ng), this indicates 200 ng is approaching the maximum detection limit for estradiol for the LC/MS/MS instrument. The accuracy of our method for extracting steroid hormones from bone ranged from 93–111%, all within acceptable values for measuring steroid hormones via LC/MS/MS (Table 2, 83.5–115.4% from Zhang et al^45^). In addition, these accuracy values are similar to studies that measured progesterone, testosterone, and hydrocortisone in gray whale blubber (88–118%),^30^ and progesterone, testosterone, and cortisol in bottlenose dolphin blubber (84–112%)^28^ via LC/MS/MS.

The matrix effect (%), or effect bone has on the ionization of hormones in the ESI source, was 113%, 100%, 104%, and 108% for progesterone, testosterone, cortisol, and estradiol, respectively. This means that there could be similar, but unknown hormones and/or hormone derivatives that positively influence target hormone concentrations. Alternatively, analyzing bone extract may result in high conservation of an analyte throughout LC/MS/MS analysis due to minor loss on the instrument's surfaces. However, final concentrations were corrected for matrix effects by addition of 100 ng of isotopically labeled steroid hormone internal standards.^29^ The recovery of extraction (%), or recovery of hormones without matrix effect, was 98%, 92%, 99%, and 101%, for progesterone, testosterone, cortisol, and estradiol, respectively. The overall process efficiency of our method for measuring hormones in walrus bone was 112%, 93%, 103%, and 110%, for progesterone, testosterone, cortisol, and estradiol, respectively. Equations used for validations are shown in Table 4.

**Table 4:** Description of sample groups (*n* = 5 each) for steroid hormone validations, as well as equations used to determine matrix effects, recovery of extraction, and process efficiency for extraction of steroid hormones from walrus bone (following Caban et al^48^)

Group	Group definition	Test	Equation used
A	Blank samples spiked pre‐extraction with hormone concentrations along calibration curve	Matrix effects	(B/A)*100
B	Bone powder extracted and spike post‐extraction with middle standard concentrations for each hormone	Recovery of extraction	(C/B)*100
C	Bone powder spiked pre‐extraction with hormone concentrations along calibration curve	Process efficiency	(C/A)*100

The percent recovery of each internal standard was calculated by comparing the ratio of mean hormone concentration detected in “Blank‐Extraction”, divided by the mean hormone concentration in the “Blank‐Dried Internal Standards” samples. The mean extraction efficiencies for each hormone in walrus bone are as follows: progesterone = 51%, testosterone = 107%, cortisol = 72%, and estradiol = 79%.

### 3.2 Measurement of steroid hormones with LC/MS/MS

**Figure 2:**
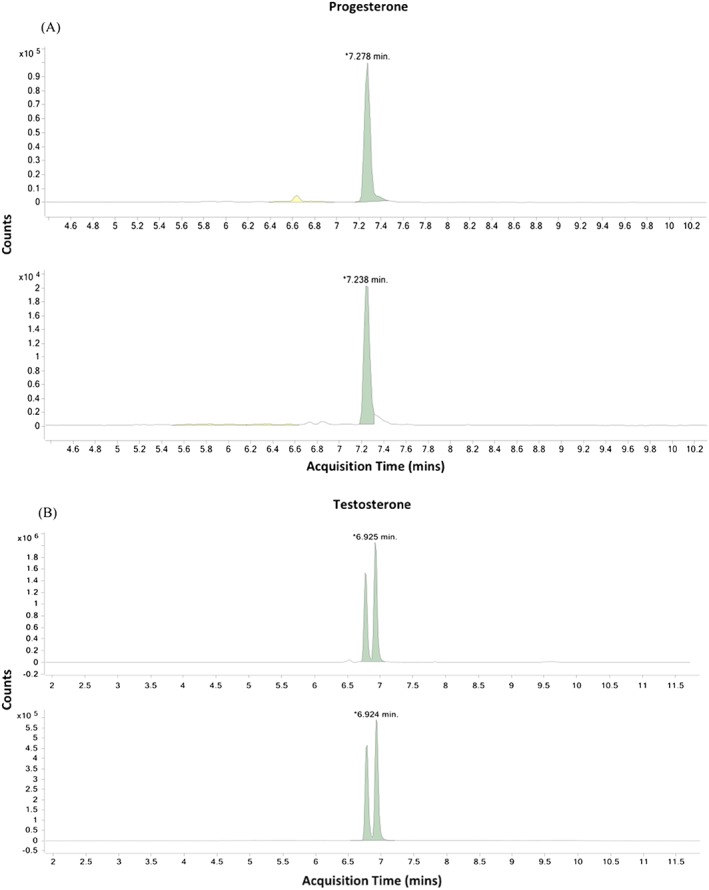
Multiple reaction monitoring chromatogram (counts) during LC/MS/MS analysis for both a dansyl chloride hormone, progesterone (A), and a keto‐derivatized hormone, testosterone (B), with acquisition times (min) labeled for a sample (top) and isotopically labeled internal standards (bottom). Internal standards correct for any sample loss during the extraction process, which explains different counts on the y‐axis. Keto derivatives resulted in a split peak in the sample and internal standard (B). The larger of the two peaks was integrated to determine hormone concentration and the other was used as a qualitative check [Color figure can be viewed at http://wileyonlinelibrary.com]

Multiple reaction monitoring was used for accurate detection of each steroid hormone. Thus, two product ions were checked for each steroid hormone using two different optimized collision energies (Table 5). Dansyl chloride derivatization provided more sensitive detection of low estradiol and the estradiol internal standard concentrations,^54^ and, thus, estradiol concentrations in walrus bone were determined by observing 506.1→171.0, 155.80 *m/z* with a resulting elution time of 7.746 min (Figure 2A). Keto derivative kits from AB Sciex increased the sensitivity for detecting and quantifying cortisol and testosterone concentrations (e.g., Star‐Weinstock et al^55^). Monitoring of 477.10→418.30, 388.20 for cortisol and 403.10→344.10, 164.00 for testosterone resulted in elution times of 6.034 min and 6.925 min, respectively (Figure 2B). The keto derivative tag for cortisol and testosterone resulted in a split double peak along the column (Figure 2B). The split peak is most likely due to the keto derivative kit used for testosterone and cortisol that creates carbon isomers that separate during LC/MS/MS analysis.^55^ For consistency, the peak with the largest magnitude of the two product ions was used to calculate steroid hormone concentrations, with the other being used as a qualitative check. A summary of product ions and source fragmentation energies optimized for detecting and measuring steroid hormones in walrus bone are provided in Table 5.

**Table 5:** List of precursor ions, two product ions, and collision energies analyzed using multiple reaction monitoring during LC/MS/MS analysis of steroid hormones and internal standards in walrus bone

Steroid hormone	Atomic mass units	Precursor ion (*m/z*)	Product ion (*m/z*)	Product ion (*m/z*)	Collision energy (V)
Progesterone	314.47	315.20	109.00	97.00	15, 15
Testosterone	288.42	403.10	344.10	164.00	20, 40
Cortisol	362.47	477.10	418.30	388.20	15, 35
Estradiol	272.38	506.10	171.00	155.80	30,40
* ^2^H_4_‐cortisol	366.49	481.10	422.30	392.30	15, 35
* ^13^C_3_‐testosterone	291.40	406.10	347.10	167.00	20, 40
* ^2^H_9_‐progesterone	323.52	324.20	113.00	100.00	15, 15
* ^2^H_5_‐estradiol	277.41	511.10	171.00	155.80	30, 40

* Indicates isotopically labeled internal standard hormone.

### 3.3 Steroid hormone concentrations in walrus tissues

#### 3.3.1 Bone

Progesterone, testosterone, cortisol, and estradiol were successfully measured in walrus bone from the archaeological, historical, and modern time periods. Concentrations of at least one order of magnitude for each hormone from each time period indicates wide variability in hormone concentrations deposited in cortical bone, which could provide insight into stress and reproductive physiology (Table 6). Of the total number of bone samples (*n* = 119, excluding analytical validation samples), ~12% of samples fell below detectable concentrations of 2.0 ng/g bone for progesterone (*n* = 14 total, n = 1 archaeological, *n* = 3 historical, and *n* = 10 modern). Estradiol was non‐detectable (ND) in ~18% of samples (*n* = 22 total, *n* = 0 archaeological, *n* = 13 historical, and *n* = 9 modern). Cortisol and testosterone peaks were detected in all samples (detected limit was 0.02 and 1.31 ng/g for cortisol and testosterone, respectively).

**Table 6:** Median (reported in ng/g lipid), standard deviation (±1 SD), mean, and range of steroid hormone concentrations measured in archaeological (> 200 years before present [bp]), historical (200–20 bp), and modern (2014–2016) walrus bone. The mean (ng/g bone, [ng/g]) is reported for reference with tissue comparison values (see Tables 5 and 6). See Appendix 1 for provenience data of samples

Sampling time period	Sample size (*n*)	Hormone	Mean ± 1 SD median (ng/g lipid)	Range: Min – Max (ng/g lipid)	Mean (ng/g)
Archaeological (> 200 bp)	10	Progesterone	3507.52 ± 9229.13 119.91	20.26–29507.71	29.46
		Testosterone	378.85 ± 513.00 202.71	103.18–1803.85	10.28
		Cortisol	143.89 ± 256.75 55.67	11.86–862.97	3.99
		Estradiol	2339.91 ± 2611.55 1701.91	10.01–7161.93	63.48
Historical (200–20 bp)	10	Progesterone	1123.76 ± 1339.61 305.16	42.16–3673.68	22.29
		Testosterone	570.82 ± 301.61 600.84	66.02–1136.22	11.32
		Cortisol	57.40 ± 66.96 39.99	7.29–239.28	1.34
		Estradiol	3149.67 ± 3285.09 3012.64	23.18–6635.50	62.47
Modern (2014–2016)	10	Progesterone	1904.33 ± 915.91 154.85	11.98–10548.32	24.51
		Testosterone	1629.58 ± 4489.00 164.43	40.36–14392.77	78.78
		Cortisol	804.98 ± 2317.79 48.29	8.42–7395.37	38.51
		Estradiol	1457.09 ± 1242.30 2055.72	20.16–2693.03	70.45

Bone serves as a long‐term reservoir for steroid hormones and lipids, but the reservoir is not metabolically inert nor do all steroid hormones biochemically behave similarly in cortical bone,^18^ which could lead to ND concentrations in bone from different time periods. Lipids marginally deteriorate in bone over time and hormones could potentially be degraded biologically and/or abiologically into more stable metabolites or leave bone completely with lipids through leaching, which could result in ND concentrations.^15^ However, archaeological bones had only one sample below detectable concentrations. Thus, hormones may remain stable inside remaining bone lipids over time, although we only measured 10 archaeological samples compared with 106 historical and modern samples, leaving a lesser chance of ND in archaeological samples. Museums tend to remove lipids from specimens for stable preservation and aesthetics,^56^ which most likely explains the low lipid content in historical bones and the number of ND historical samples (*n* = 16) that came from museum collections. Modern animals accounted for the majority of ND samples (total *n* = 19, progesterone *n* = 10, estradiol *n* = 9). Of the modern ND samples (total n = 19), males had ND concentrations of progesterone (*n* = 6 of 10) and estradiol (n = 1 of 9). Progesterone and estradiol would be expected to be low in male walruses, because progesterone and estradiol are female reproductive hormones and only play a minor role in male reproduction.^24^, ^57^ In females, estradiol does not remain elevated for extended periods of time in pregnant walruses compared with progesterone, which may stay elevated for up to 9 months throughout a pregnancy.^7^, ^58^, ^59^ Thus, surges of estradiol are more difficult to capture in a long‐term reservoir tissue, such as bone, compared with progesterone and could result in ND samples for modern female walrus bone samples (*n* = 8 of 10). While estradiol concentrations are discussed in more detail below, estradiol can be locally synthesized in bone,^18^, ^60^ and most likely has a different reservoir time in bone compared to the other steroid hormones in this study, which could contribute to the relatively high number of ND samples. The modern female samples with below detectable concentrations of estradiol were all collected between the months of April to May, which is outside the timeframe when female walruses would go into estrus and have high estradiol concentrations.^51^, ^58^ Additionally, one of the animals was a juvenile. Testosterone and cortisol were above detectable limits in all bone samples. Possibly, testosterone and cortisol are more prone to deposition in cortical bone, do not degrade at a similar rate, or methanol was more effective at extracting testosterone and cortisol compared with progesterone and estradiol. Additionally, new data suggest different hormone metabolites could be deposited into different tissues of marine mammals.^61^ In blue whale (Balaenoptera musculus) feces, two different stress hormones were measured and results indicated corticosterone was the dominant stress hormone deposited in feces compared to cortisol.^61^ Additional LC/MS/MS testing could confirm testosterone and cortisol as the preferred deposited metabolites for those hormones in cortical bone, and if other hormone metabolites of progesterone and estradiol are being deposited into bone.^28^ However, progesterone and estradiol should be the targeted hormones when studying walrus reproductive physiology, because they were detected in the majority of samples and are the main hormones that initiate and sustain the female reproductive cycle in walruses.^58^

#### 3.3.2 Serum and blubber

In blubber, all 2015 male samples, excluding one duplicate, were below detectable limits for progesterone (*n* = 19, total 2015 male blubber samples *n* = 20). Estradiol in blubber from 2015 was below detectable limits in 20 of 22 samples (including duplicates from one 2015 female). From our results, estradiol is most likely not deposited in the blubber layer in high concentrations, possibly due to having relatively low circulating concentrations to begin with in male and female pinnipeds.^57^, ^58^ Female walruses usually enter estrus twice in the year, around January and late August.^51^ Estradiol increases in female pinnipeds before entering estrus,^62^ but all modern females used for the tissue comparison were collected in May, a time of low circulating estradiol concentrations (Appendix 3). Additionally, surges in estradiol have not always been documented in walruses before estrus, but instead may be more sporadic.^58^ Hence, in 2015 there were most likely no surges in estradiol to detect in blubber or serum (Table 7). Estradiol has been measured in the blubber of gray whales (Eschrichtius robustus) by the use of EIAs with detectable concentrations in male calves, juveniles, adults, and adult females.^63^ However, there were lower concentrations (max ~0.5 ng/g of blubber) detected in samples with greater mass (100–200 mg).^63^ Our sample masses were higher, 200–300 mg of blubber, and we used LC/MS/MS for detecting hormone concentrations which would most likely lead to lower estradiol concentrations in walrus blubber compared with those sampled in Mello et al^63^ that used lower sample mass and EIAs to detect estradiol. LC/MS/MS analysis of three gray whale (Eschrichtius robustus) blubber samples resulted in progesterone being ND in a juvenile male (*n* = 1), while testosterone was ND in one adult female (n = 1).^30^ In bottlenose dolphin (*Tursiops truncates*) blubber samples analyzed with LC/MS/MS, cortisol, progesterone, and testosterone were detectable in all samples (*n* = 4 total, female = 2, male = 2).^28^ Progesterone was detectable in the two male dolphins, though concentrations were low (0.473 and 0.262 ng/g).^28^ ND or low concentrations of progesterone in males are similar to our results (Table 8). Further, all serum samples had detectable concentrations of all steroid hormones analyzed. While measuring these hormones in plasma may provide less error, our results of detectable concentrations of all hormones in serum samples are similar to those measured in dolphin serum using LC/MS/MS methods.^36^

**Table 7:** Mean estradiol concentrations (ng/for male and female walrus tissues (i.e., bone, blubber, and serum) ± 1 SD, median concentrations in ng/g lipid (for reference purposes only), concentration ranges (non‐lipid corrected), and sample sizes (n). Walruses were harvested in 2014 and 2015 by Native subsistence hunters on St Lawrence Island, AK, USA. Significant differences among mean log‐transformed estradiol concentrations are indicated with bolded *P*‐values from three‐way ANOVAs testing differences using two main factors (i.e., sex and tissue) and an interaction term (i.e., sex*tissue) for separate sampling years. If the tissue factor or the interaction term was significant, relevant *P*‐values (bolded if significant) from the Tukey post hoc tests are reported

Year	Sex	Hormone	Tissue [units]	Sample size (n)	Mean ± 1 SD median [ng/g lipid]	Range (min ‐ max)	** *P*‐value (year)	*P*‐value (sex)	*P*‐value (tissue)	*P*‐value (tissue, Tukey post hoc)	*P*‐value (sex*tissue)
2014	Males	Estradiol	Bone [ng/g]	18	118.85 ± 20.69 2387.16	100.31–194.85	**<0.001**	0.06	**<0.001**	(blubber, 0.51) (**serum**, **<0.001**)	0.96
			Blubber [ng/g]	17	124.73 ± 16.74	106.93–171.88				(bone, 0.51) (**serum**, **<0.001**)	
			Serum [ng/mL]	8	98.83 ± 25.34	83.57–160.44				(**bone**, **<0.001**) (**blubber**, **<0.001**)	
	Females	Estradiol	Bone [ng/g]	5	114.78 ± 8.82 2383.13	100.57–124.14	**<0.001**	0.06	**<0.001**	(blubber, 0.51) (**serum**, **<0.001**)	0.96
			Blubber [ng/g]	4	118.29 ± 6.71	112.96–127.60				(bone, 0.51) (**serum**, **<0.001**)	
			Serum [ng/mL]	5	91.89 ± 6.39	82.91–99.75				(**bone**, **<0.001**) (**blubber**, **<0.001**)	
2015	Males	Estradiol	Bone [ng/g]	10	1.43 ± 0.96 23.96	0.65–4.01	**<0.001**	‐	0.38	‐	‐
			Blubber [ng/g]	10	1.21 ± 0.51	0.89–2.35				‐	‐
			Serum [ng/mL]	8	1.83 ± 1.28	0.77–4.60				‐	‐
	Females	Estradiol	Bone [ng/g]	1	1.44 *29.79	‐	**<0.001**	‐	0.38	‐	‐
			Blubber [ng/g]	1	0.97	‐				‐	‐
			Serum [ng/mL]	1	1.34	‐				‐	‐

* Due to only one female, value represents her estradiol concentrations [ng/g bone] in [ng/g lipid].

** Because “Year” was significant, log transformed estradiol concentrations were analyzed via ANOVAs seperately by year for differences among tissues and between sexes.

**Table 8:** Mean hormone concentrations (i.e., cortisol, progesterone, and testosterone) for male and female walrus tissues (i.e., bone, blubber, and serum) ± 1 SD, median concentrations in ng/g lipid (reference purposes only), concentration ranges (non‐lipid corrected), and sample sizes (n). samples came from 2014–2015 native harvests on St. Lawrence Island, AK. Significant (bolded) *P*‐values are from the three‐way ANOVAs testing differences among mean log transformed steroid hormone concentrations using two main factors (sex and tissue) and an interaction term (sex*tissue). If the tissue factor or the interaction term was significant, relevant *P*‐values (bolded if significant, α < 0.05) from the Tukey post hoc tests are reported. Only female blubber progesterone concentrations were significantly different compared with male walrus tissues relating to the interaction term (sex*tissue), thus only those significant *P*‐values are reported. The second set of *P*‐values for progesterone are results of repeated ANOVA as previously mentioned, but without the 2014 pregnant female walrus potentially skewing initial ANOVA progesterone results

Sex	Tissue [units]	Sample size (n)	Hormone	Mean ± 1 SD median [ng/g lipid]	Range (min – max)	*P*‐value (sex)	*P*‐value (tissue)	*P*‐value (tissue, Tukey post hoc)	*P*‐value (sex*tissue)	*P*‐value (sex*tissue, Tukey post hoc)
Males	Bone [ng/g]	28	Cortisol	9.78 ± 22.51 67.15	0.22–118.84	0.06	**<0.001**	(blubber, 0.96) (**serum**, **<0.001**)	0.14	‐
			Progesterone	31.67 ± 57.35 193.59	0.17–264.20	**(<0.001, 0.002)**	(0.27, 0.28)	‐	**(0.009, 0.04)**	(male:Bone*female:Blubber, **0.007, 0.04**)
			Testosterone	13.25 ± 11.53 255.20	2.18–64.48	1.0	**0.005**	(**blubber**, **0.003**) (serum, 0.26)	0.75	‐
	Blubber [ng/g]	27	Cortisol	4.35 ± 3.31	0.66–13.17	0.06	**<0.001**	(bone, 0.96) (**serum**, **<0.001**)	0.14	‐
			Progesterone	4.47 ± 3.58	0.89–15.71	**(<0.001, 0.002)**	(0.27, 0.28)	‐	**(0.009, 0.04)**	(male:Blubber*female:Blubber, **<0.001, 0.003**)
			Testosterone	8.17 ± 7.01	0.54–24.62	1.0	**0.005**	(**bone**, **0.003**) (serum, 0.33)	0.75	‐
	Serum [ng/mL]	16	Cortisol	20.80 ± 7.07	10.68–33.39	0.06	**<0.001**	(**bone**, **<0.001**) (**blubber**, **<0.001**)	0.14	‐
			Progesterone	5.45 ± 5.40	0.92–20.46	**(<0.001, 0.002)**	0.27	‐	**(0.009, 0.04)**	(male:Serum*female:Blubber, **0.0011, 0.009**)
			Testosterone	8.50 ± 3.05	4.96–14.79	1.0	**0.005**	(bone, 0.26) (blubber, 0.33)	0.75	‐
Females	Bone [ng/g]	6	Cortisol	4.29 ± 6.25 46.35	0.73–16.90	0.06	**<0.001**	(blubber, 0.96) (**serum**, **<0.001**)	0.14	‐
			Progesterone	12.67 ± 5.80 205.72	6.18–21.31	**(<0.001, 0.002)**	(0.27, 0.28)	‐	**(0.009, 0.04)**	**Reported above**
			Testosterone	16.72 ± 19.62 188.72	5.25–56.21	1.0	**0.005**	(**blubber**, **0.003**) (serum, 0.26)	0.75	‐
	Blubber [ng/g]	5	Cortisol	6.15 ± 2.65	2.42–8.55	0.06	**<0.001**	(bone, 0.96) (**serum**, **<0.001**)	0.14	‐
			Progesterone	85.50 ± 45.44	21.23–141.98	**(<0.001, 0.002)**	(0.27, 0.28)	‐	**(0.009, 0.04)**	**Reported above**
			Testosterone	8.26 ± 5.74	1.02–16.79	1.0	**0.005**	(**bone**, **0.003**) (serum, 0.33)	0.75	‐
	Serum [ng/mL]	6	Cortisol	49.41 ± 24.89	23.35–80.37	0.06	**<0.001**	(**bone**, **<0.001**) (**blubber**, **<0.001**)	0.14	‐
			Progesterone	7.22 ± 5.54	3.24–18.04	**(<0.001, 0.002)**	(0.27, 0.28)	‐	**(0.009, 0.04)**	**Reported above**
			Testosterone	6.88 ± 2.95	2.87–10.61	1.0	**0.005**	(bone, 0.26) (blubber, 0.33)	0.75	‐

### 3.4 Walrus tissue comparison

Tissues were compared to determine if bone steroid hormones were similar to serum or blubber hormone concentrations and to gauge whether there might be a different reservoir time in bone compared to the other tissues. This comparison is different than the bone physiological validations in that the physiological validations can determine a more accurate reservoir time of steroid hormones in bone based on reproductive status comparisons. The tissue comparison results and discussion only include the animals that had two or more tissues from 2014–2015 (Appendix 3, *n* = 32 individuals).

#### 3.4.1 Cortisol

Mean cortisol concentrations were significantly different among tissues (*P* < 0.001). Serum cortisol concentrations were significantly higher than walrus blubber and bone (Tukey's post hoc test, *P* < 0.001, < 0.001, respectively), while blubber and bone cortisol concentrations were similar (*P* = 0.96). Blubber cortisol concentrations are potentially a longer accumulated measure compared with serum in pinnipeds,^10^ which is likely the reason why blubber cortisol concentrations are not significantly different from bone cortisol concentrations (Table 8). This lends further support to the idea that bones are a long‐term reservoir of steroid hormones with the possible exception of estradiol, and can be used to monitor long‐term stress response that will not be skewed by acute stressors.^20^ With the slow bone turnover rate of 3% cortical bone/year,^19^ cortical bone might even be a longer‐accumulated average of steroid hormone concentrations than the weekly to monthly average of blubber.^10^

#### 3.4.2 Estradiol

Estradiol concentrations were significantly different between years (ANOVA, *P* < 0.001, Table 7). Thus, 2014 samples were tested for estradiol concentration differences among tissues, between sexes, and the interaction of sex and tissue sampled (Table 7). Samples from 2014 had similar mean estradiol concentrations between sexes with no effect of the interaction of sex and tissue (*P* = 0.06, 0.96, respectively), but concentrations were significantly different among tissues (*P* < 0.001). Bone and blubber estradiol concentrations were similar (Tukey's post hoc test, *P* = 0.51), but both were significantly higher than serum for 2014 samples (*P* < 0.001, < 0.001, respectively). Samples from 2015 only contained one female, thus only differences among tissues were tested (Table 7). In contrast to the 2014 samples, the samples from 2015 had similar concentrations among tissues (*P* = 0.38). Bone turnover rate is slow and hormone concentrations potentially represent a long‐term accumulated average for a walrus. However, estradiol is also an important hormone in stimulating bone turnover, helping to increase bone mineral density, and is locally produced in bone.^16^, ^18^, ^64^ It is still unknown how much this local production of estradiol contributes to overall estradiol concentrations compared with gonadal production, but bone is still an estradiol reservoir to some degree.^18^ Therefore, estradiol may not have similar long‐term reservoir times compared with other hormones measured in this study.

#### 3.4.3 Progesterone

Mean progesterone concentrations were significantly different between sexes (ANOVA, *P* < 0.001) and the interaction of sex and tissue (*P* = 0.009), but not tissue as a main effect (*P* = 0.27). Female blubber progesterone concentrations were driving the significant differences seen in the interaction term (i.e., sex*tissue). Female blubber progesterone concentrations were significantly higher compared with male blubber, bone, and serum progesterone concentrations (Tukey's post hoc test, *P* < 0.001, 0.007, 0.001, respectively). Bone progesterone concentrations for females were lower than blubber, but higher than serum progesterone concentrations (Table 8). Females were adults (except for one unknown), therefore the blubber progesterone is expected to be high due to prolonged circulating progesterone concentrations related to the preceding breeding season.^21^ This is especially true for the one pregnant female walrus, which had the highest measured concentrations (141.98 ng/g) in blubber. Only bone was available for female subadults, and thus subadults were only included in the physiological validations and not tissue comparison (see section 2.10 for age class breakdown). Males in the tissue comparison had significantly higher progesterone concentrations in bone compared with females (Table 8). Progesterone is not only the main female pregnancy hormone, but is also a precursor to other important reproductive (i.e., estradiol and testosterone) and stress (i.e., cortisol) steroid hormones.^29^ As mentioned, bone steroid hormone concentrations are a long‐term accumulated average of a walrus based on the slow cortical bone turnover rate (3% /year).^19^ Male walruses potentially could use cortical bone as a reservoir for progesterone to be metabolized by the metabolically active bone marrow into other important hormones when needed.^16^, ^18^ For example, in rats, stress can reduce circulating testosterone concentrations, but when injected with biologically high progesterone concentrations, male reproductive behavior occurred despite low circulating testosterone.^65^

#### 3.4.4 Testosterone

Mean testosterone concentrations were significantly different among tissues (ANOVA, *P* = 0.005), but not between sexes (*P* = 1.0), nor the interaction of sex and tissue (*P* = 0.75). Significant differences among walrus tissues were only found among bone and blubber testosterone concentrations (Tukey' post hoc test, *P* = 0.003), but not among serum and blubber (*P* = 0.33) or serum and bone (*P* = 0.26). Bone testosterone concentrations among walruses showed higher levels compared with serum and blubber (Table 8). Adult walruses are the only well‐represented age class, therefore higher testosterone concentrations in adult male bone could reflect the accumulated average of numerous breeding seasons (Table 8). Females had relatively high bone testosterone concentrations compared with males (Table 8). In females, elevated fecal testosterone concentrations have been associated with pregnancy and dominance behavior in wild hybrid baboons (*Papio* spp.).^66^ In human females, elevated saliva testosterone concentrations during the estrus cycle correlated with an increase in attractiveness to males.^67^ Similar to males, females in this study were adults, and higher testosterone in bone compared with serum and blubber could indicate older dominant reproductive females. All females, except one, were either accompanied by a calf and/or yearling, lactating, or pregnant, indicating that they were sexually mature (Appendix 3). Testosterone is also known to be an important hormone for conversion into estradiol, which helps stimulate bone turnover in humans;^64^ however, this was not clearly demonstrated in rats.^18^ Further research into how testosterone is converted into estradiol in walrus bone is needed to determine the role bone turnover has on testosterone concentrations in walruses.

### 3.5 Physiological validations of steroid hormones in walrus bone

Based on female physiological validations, steroid hormone concentrations in cortical bone represent a long‐term accumulated average reservoir. Pregnant females had similar progesterone, cortisol, and testosterone concentrations compared with non‐pregnant adult females (Kruskal‐Wallis, *P* ≥ 0.05 for all adult females regardless of reproductive status, Table 3A). Based on previous studies, progesterone and cortisol concentrations should be significantly higher in pregnant females compared with non‐pregnant females, if bone steroid hormones are indicative of an acute reproductive event, such as pregnancy,^2^, ^21^, ^22^, ^62^, ^68^ yet we did not observe this in the known pregnant vs. known non‐pregnant females (Table 3A). We did see high variability in progesterone, testosterone, cortisol, and estradiol concentrations, which could indicate the reproductive success of individuals (Table 3A). That is, expected accumulation of progesterone should be higher in the bone of a female that has had three pregnancies compared with a female of the same age that has had only one pregnancy. Our results of similar progesterone concentrations in pregnant and non‐pregnant adult females also suggest steroid hormones in cortical bone represent a long‐term reservoir, most likely greater than the 15 month timeframe based on the long gestation and elevated progesterone concentrations in serum of pregnant walruses.^51^, ^58^ Pregnant and lactating females had similar median cortisol concentrations compared with known non‐pregnant females (Table 3A). Pregnant female marine mammals typically have significantly higher cortisol concentrations compared with non‐pregnant females.^4^, ^36^, ^69^ However, in pinnipeds, elevated serum cortisol concentrations have been observed during late pregnancy, with peak cortisol concentrations documented during lactation.^9^, ^70^, ^71^, ^72^ Thus, if bone concentrations detected short‐term elevations of cortisol, we would expect significantly higher cortisol concentrations in pregnant and lactating females compared with non‐pregnant females. However, non‐pregnant females had similar concentrations to both lactating and pregnant females (Table 3A). Perhaps the slow turnover of cortical bone does not integrate elevated cortisol concentrations of pregnant or lactating walruses into the bone quickly enough to make a distinction among non‐pregnant females (Table 3A), indicating cortical bone serves as a long‐term reservoir of cortisol.

Pregnant walruses did have significantly higher estradiol concentrations compared with non‐pregnant adult females (*P* = 0.03, 0.01, respectively, Table 3B). However, pregnant females had similar estradiol concentrations to non‐pregnant females that were lactating and/or were accompanied by offspring. Estradiol is unique in bone tissue, because it is an important component in maintaining bone mineral density in both males and females.^64^ In addition, estradiol can be locally synthesized in bone by the aromatization of testosterone.^18^ Thus, estradiol concentrations measured in walrus bone probably turn over on a shorter time scale, e.g., seasonally, unlike the other steroid hormone concentrations measured in this study (Table 3B). The apparent shorter reservoir time could explain why estradiol was significantly different between pregnant and non‐pregnant females (Table 3B). While the non‐pregnant females that were lactating and/or were accompanied by offspring did not contain a fetus, if they had a calf, there is a possibility they would have recently given birth and, hence, had similar estradiol concentrations compared with pregnant females (Table 3B).

There were ample subadult female bones for the physiological validation analyses compared with the tissue comparison test (*n* = 18 and *n* = 0, physiological validations and tissue comparison, respectively). Subadult females had significantly higher steroid hormone concentrations compared with adult females, with the exception of cortisol measured in known non‐pregnant females (Tables 3A and 3B). There are a couple of possibilities why subadults had higher hormone concentrations compared with the adult females. The majority of these subadult females (*n* = 15 of 18 total subadult females) were from the 1950s, 1960s, and 1970s, a time of rapid population increase,^73^, ^74^ age of maturation was lower (approximately 8 years old compared to 10 years old in the 1980s),^73^ and fecundity in females was higher during the 1950s to 1970s due to low population numbers and abundant resources that allowed for a population increase.^73^, ^75^, ^76^ Finally, because age of maturation can shift in walruses and other Arctic pinnipeds,^51^, ^73^, ^75^, ^77^, ^78^ it is possible that age classes assigned did not reflect reproductive maturity.

Subadult males (3–14 years)^51^ did not have significantly different testosterone concentrations compared with adult males (15–28 years old,^51^ *P* = 0.34, Table 3A). In previous studies of male spotted seals (Phoca largha) and a male walrus, testosterone concentrations were higher in mature males.^57^, ^79^ If bone testosterone was higher in adults compared with subadults, this would be an indication of a short‐term reservoir time of testosterone in cortical bone.^8^, ^57^ Additionally, the similarity of subadults and adults could be an indication of harem‐style breeding typical for walruses, with few dominant reproductive males, who would have higher testosterone concentrations, and a majority of subordinate males with lower testosterone levels similar to subadult males.^51^ However, male rut was induced in a captive male adult walrus resulting in a peak serum testosterone concentration of 12.59 ng/ mL.^59^ Taking into account that RIAs were used to measure these hormones, possibly inflating the concentration due to cross reactivity, by comparison, our maximum serum testosterone concentration measured in wild walruses using LC/MS/MS was 14.79 ng/mL. Our maximum bone testosterone concentration for males was 64.58 ng/g. Overall, testosterone in cortical bone is not affected by age class and concentrations are four times that of the maximum testosterone concentration in serum, lending evidence to cortical bone as a long‐term reservoir of testosterone.

## 4 CONCLUSIONS

This is the first study to develop a method for extracting, measuring, and quantifying progesterone, testosterone, cortisol, and estradiol concentrations in walrus bone as old as 3585 bp using LC/MS/MS. The multiple reaction monitoring combined with the positive ESI mode during the LC/MS/MS analysis provided the best results, when detecting hormones extracted from bone. Dansyl chloride and keto derivatizations increased the sensitivity of the LC/MS/MS instrument providing a higher number of detectable signals for steroid hormones from bone tissue that has low concentrations of steroid hormones based on their lipid content. Steroid hormones measured in bone were validated for linearity, accuracy, matrix effects, precision, and extraction efficiencies, with all values falling within acceptable published ranges.

Physiological validation and tissue comparison analyses revealed that steroid hormones in bone represent a long‐term reservoir time (possibly 10–20 years). Our results are also consistent with bone steroid hormone concentrations representing a long‐term reservoir of steroid hormones compared with serum, and similar to blubber, meaning hormone concentrations in bone are not skewed by “short‐term” reproductive events with the exception of estradiol.^60^ The tissue comparison showed that progesterone and cortisol concentrations measured in bone are not similar to serum, but similar to blubber, meaning bone may have a longer‐term reservoir for these hormones compared with serum (Tables 7 and 8, Appendix 2). Testosterone in bone and serum was similar in the tissue comparison; however, physiological validations among males show that immature and mature males had similar bone testosterone concentrations and bone had over four times the maximum concentration of testosterone compared with serum, which could indicate bone as a longer‐term reservoir of testosterone. Serum and blubber represent approximately hourly to monthly reservoir times of steroid hormones, respectively.^21^, ^37^ Thus, serum and blubber steroid hormone concentrations are affected by a singular reproductive season,^4^, ^10^, ^21^, ^68^ but our physiological validations indicate bone steroid hormone concentrations (except estradiol) are not affected by a single reproductive event (Table 3A). Combining our results of the tissue comparison and the physiological validation, steroid hormone concentrations measured in cortical bone represent a long‐term reservoir of steroid hormones (Tables 3A, 3B, 5 and 6). This agrees with Yarrow et al,^18^ who suggested there are reservoirs of estrogens and androgens in rat bones; however, they did not suggest a timeframe for that reservoir. Steroid hormones are lipophilic, and lipids in bone are associated with cortical bone cells and its mineralized tissue.^16^, ^80^ There has been evidence of a strong positive linear relationship of bone cell turnover and lipid accumulation in rat bone.^81^ While not directly transferrable to walruses, the relationship between bone cell turnover and lipid accumulation in bone supports our suggestion that steroid hormones, being lipid‐associated molecules, have a slow turnover rate in walrus cortical bone (~3%/year).^19^ Thus we posit, based on the results from the physiological validations, tissue comparisons, and published literature on bone physiology, that steroid hormones (progesterone, testosterone, and cortisol) measured in adult walrus cortical bone represents an accumulated average over a 10–20‐year time period (3% cortical bone/year for humans^19^ translates to ~33 years complete turnover of cortical bone in walrus with 10–20 years being conservative, see full calculation in Charapata^60^). We should note this does not apply to all steroid hormones, most notably estradiol, which may be quite different due to its local production in bone, possibly with a seasonal or yearly turnover.

Ecological studies using bone steroid hormones would be most applicable for monitoring long‐term physiological changes in animal populations. For example, this method can shed light on walrus physiology in response to a rapidly changing Arctic ecosystem by comparing modern animals experiencing sea ice loss to archaeological and historic walruses during differing climate regimes. As bone is one of the few tissues surviving for millennia, our method is ideal to put present ecosystem change into context, where no other tissues remain that could provide a true reference point for comparison with modern walrus physiology.

## APPENDIX 1

List of walrus specimens from archaeological (greater than 200 years before present [BP]), historical (20–200 BP), and modern (2014–2016) time periods with their respective provenience and steroid hormone concentrations data. Samples were collected from various archaeological collections from the University of Alaska (UAM: ARCH), Sanak Island excavation (Sanak), and collections curated by Dr. A. Jensen at Ukpeaġvik Iñupiat Corporation (Utqiaġvik). Historical samples came from mammology collections from University of Alaska (UAM: MAMM) and the National Institute of Natural History (Smith: Mamm). Modern samples were collected by Native subsistence hunters on St. Lawrence Island, Alaska (Subsistence). Samples with ranges of calibrated dates were determined by using the earliest and latest radiocarbon dates respective to the archaeological excavation, where the walrus bone was found. *Indicates collected dates determined by Atomic Mass Spectrometry (AMS) radiocarbon dating techniques (± Error). Lipid and non‐lipid corrected steroid hormone concentrations (ng/g lipid and ng/g bone, respectively) abbreviated as follows: progesterone: (P), testosterone (T), cortisol (C), and estradiol (E).

**Table:**

WAL ID	Catalog number	Source	Sex	Year collected	Median calibrate before present (BP)	Element	Location	Age class	Tissue	Sample time period	P (ng/g lipid)	T (ng/g lipid)	C (ng/g lipid)	E (ng/g lipid)	P (ng/g powder)	T (ng/g powder)	C (ng/g powder)	E (ng/g powder)
WAL023.1	16588	UAM: Mamm	Female	1933	‐	Mandible	St. Lawrence	Adult	Bone	Historical	281.04	586.16	57.13	5964.97	5.57	11.63	2.13	118.31
WAL033.1	10538	UAM: Mamm	Female	1973	‐	Mandible	Kotzebue	Adult	Bone	Historical	1727.08	808.36	43.57	6373.37	34.25	16.03	1.32	126.41
WAL046.1	14793	UAM: Mamm	Male	1981	‐	Mandible	Port Moller	Adult	Bone	Historical	265.66	692.51	14.68	6213.04	5.27	13.74	1.89	123.23
WAL048.1	11702	UAM: Mamm	Female	1956	‐	Skull	St. Lawrence	Adult	Bone	Historical	3673.68	1136.22	69.98	6635.50	72.86	22.54	0.57	131.61
WAL069.1	16586	UAM: Mamm	Female	1933	‐	Mandible	St. Lawrence	Adult	Bone	Historical	329.29	668.37	28.98	6122.91	6.53	13.26	0.32	121.44
WAL140.1	S14–0027	Subsistence	Female	2014	‐	NA	Savoonga	Adult	Bone	Modern	1526.27	722.84	63.37	2483.80	73.79	34.95	3.06	120.08
WAL144.1	S14–0036	Subsistence	Male	2014	‐	NA	Savoonga	Adult	Bone	Modern	2753.11	328.88	60.23	2634.24	133.10	15.90	2.91	127.36
WAL149.1	S14–0045	Subsistence	Female	2014	‐	NA	Savoonga	Adult	Bone	Modern	1269.63	142.47	15.66	2370.49	17.02	6.89	0.76	114.61
WAL151.1	G14–0002	Subsistence	Female	2014	‐	NA	Gambell	Adult	Bone	Modern	1077.88	234.97	349.71	2469.19	13.72	11.36	16.91	119.38
WAL158*	VL112	Sanak	Unknown	‐	3585	Mandible	Sanak Island	Subadult	Bone	Archaeological	34.04	149.88	11.86	4369.93	0.92	4.07	2.06	118.55
WAL163*	VL129	Sanak	Unknown	‐	927	NA	Sanak Island	Unknown	Bone	Archaeological	57.62	103.18	862.97	4141.97	1.56	2.80	23.41	112.36
WAL170	SL2‐CQDQL	Utqiaġvik	Unknown	‐	500–150	Rib	Pingasagruk	Unknown	Bone	Archaeological	1390.73	1803.85	167.30	7161.93	37.73	48.93	4.54	194.29
WAL174	UA72–060‐0042	UAM: ARCH	Unknown	‐	1039–1381	Mandible	Gambell	Unknown	Bone	Archaeological	8740.18	501.54	112.01	4196.48	237.10	13.61	3.04	113.84
WAL178	UA72–065‐0527	UAM: ARCH	Unknown	‐	1450–850	Mandible	Kitnigipaluk	Adult	Bone	Archaeological	231.32	153.55	24.93	3311.33	6.28	4.17	0.68	89.83
WAL181	UA72–065‐0530	UAM: ARCH	Unknown	‐	1450–850	Mandible	Kitnigipaluk	Adult	Bone	Archaeological	47.87	615.54	7.29	25.92	0.95	12.21	0.45	0.51
WAL216	USNM63302	Smith: Mamm	Male	1895	‐	Skull	St. Paul Island	Adult	Bone	Historical	44.51	66.02	36.41	23.18	0.88	1.31	0.31	0.46
WAL227.2	USNM500254	Smith: Mamm	Male	1973	‐	Skull	St. Lawrence Island	Adult	Bone	Historical	42.16	207.61	20.88	31.93	0.84	4.12	0.57	0.63
WAL229.1	USNM16437	Smith: Mamm	Male	1880	‐	Skull	Plover Bay	Adult	Bone	Historical	15.33	186.39	72.03	29.47	0.74	9.01	1.06	1.42
WAL234	S15–039	Subsistence	Male	2015	‐	NA	Savoonga	Adult	Bone	Modern	20.26	233.10	71.61	44.04	0.55	6.32	1.08	1.19
WAL254*	UA75–009‐‐XPH‐00001	UAM: ARCH	Unknown	‐	172 ± 22	Fibula	Point Hope	Unknown	Bone	Archaeological	90.62	261.84	38.87	32.80	2.46	7.10	1.10	0.89
WAL255*	UA75–009 ‐‐ XPH‐001	UAM: ARCH	Unknown	‐	300 ± 22	Rib	Point Hope	Unknown	Bone	Archaeological	53.85	172.31	59.46	38.16	1.46	4.67	1.48	1.04
WAL263*	UA75–009 ‐‐ XRH‐00001	UAM: ARCH	Unknown	‐	300 ± 22	Bulae	Point Hope	Unknown	Bone	Archaeological	119.15	152.77	51.87	10.01	3.23	4.14	0.58	0.27
WAL271*	UA75–009‐‐XPH‐00001	UAM: ARCH	Unknown	‐	185 ± 22	Ulna	Point Hope	Unknown	Bone	Archaeological	120.66	256.51	37.99	92.49	3.27	6.96	1.95	2.51
WAL284	G15–015	Subsistence	Male	2015	‐	NA	Gambell	Adult	Bone	Modern	11.98	68.77	36.35	20.16	0.58	3.32	0.09	0.97
WAL622	2014_W_24	Subsistence	Female	2014	‐	Mandible	Utqiaġvik	Adult	Bone	Modern	76.30	14392.77	7395.37	2693.03	3.69	695.84	357.54	130.20
WAL623	2014_W_25	Subsistence	Female	2014	‐	Mandible	Utqiaġvik	Adult	Bone	Modern	13.47	76.68	20.21	1740.94	0.65	3.71	0.98	84.17
WAL769	129361	UAM: Mamm	Female	1983	‐	Mandible	Chukchi Sea (Russian side)	Adult	Bone	Historical	1971.63	407.22	55.77	45.56	39.11	8.08	1.11	0.90
WAL804	S16–034	Subsistence	Male	2016	‐	NA	Savoonga	Adult	Bone	Modern	20.46	40.36	8.42	23.80	0.99	1.95	0.41	1.15
WAL808	S16–039	Subsistence	Male	2016	‐	NA	Savoonga	Adult	Bone	Modern	17.44	101.62	28.42	105.77	0.84	4.91	1.37	5.11
WAL817	130627	UAM: Mamm	Male	1952	‐	Baculum	Gambell	Adult	Bone	Historical	2854.69	520.20	239.28	60.31	56.62	10.32	4.75	1.20

## APPENDIX 2

### 2.2

All modern and historical walrus bone samples used for the physiological validations with their respective project number from this study, catalog number from their respective source (Subsistence hunters or University of Alaska Museum [UAM: MAMM]), provenience data that was retrieved from either hunter observations or collection notes from UAM, estimated age, and their respective hormone concentrations in ng/g lipid. Estimated ages were based on counting cementum growth layers in the walrus teeth.

**Table:**

Project number	Catalogue number	Source	Sex	Year collected	Location	Age class	Age	Pregnant	Offspring present?	Lactating?	Sample type	Progesterone (ng/g lipid)	Testosterone (ng/g lipid)	Cortisol (ng/g lipid)	Estradiol (ng/g lipid)
WAL018.2	11519	UAM: MAMM	Female	1961	St. Lawrence	Subadult	‐	‐	‐	‐	Historical	3359.16	2649.88	1010.87	6922.15
WAL026	11521	UAM: MAMM	Female	1965	St. Lawrence	Subadult	‐	‐	‐	‐	Historical	8069.74	2549.52	435.03	5468.22
WAL028	11514	UAM: MAMM	Female	1956	St. Lawrence	Subadult	‐	‐	‐	‐	Historical	8686.11	3002.19	164.64	6878.43
WAL029	11513	UAM: MAMM	Female	1956	St. Lawrence	Subadult	‐	‐	‐	‐	Historical	8665.99	2445.95	2638.75	9256.06
WAL030.1	11515	UAM: MAMM	Female	1956	St. Lawrence	Subadult	‐	‐	‐	‐	Historical	7717.21	2741.71	1050.27	6271.99
WAL035	11682	UAM: MAMM	Female	1958	St. Lawrence	Subadult	‐	‐	‐	‐	Historical	7006.50	2629.83	88.40	7874.43
WAL053	11698	UAM: MAMM	Female	1956	St. Lawrence	Subadult	‐	‐	‐	‐	Historical	14409.69	6367.67	317.79	5786.77
WAL054	11685	UAM: MAMM	Female	1957	St. Lawrence	Subadult	‐	‐	‐	‐	Historical	17892.16	7396.98	353.39	8089.23
WAL056.1	11637	UAM: MAMM	Female	1959	St. Lawrence	Subadult	‐	‐	‐	‐	Historical	6279.25	1077.95	100.99	7692.44
WAL057.2	11473	UAM: MAMM	Female	1956	St. Lawrence	Subadult	‐	‐	‐	‐	Historical	448.79	641.17	66.62	6567.48
WAL063.1	11518	UAM: MAMM	Female	1966	St. Lawrence	Subadult	‐	‐	‐	‐	Historical	6243.97	3393.02	238.42	8522.71
WAL065.1	11696	UAM: MAMM	Female	1957	St. Lawrence	Subadult	‐	‐	‐	‐	Historical	3063.16	1268.59	95.30	6207.78
WAL081.1	11704	UAM: MAMM	Female	1958	St. Lawrence	Subadult	‐	‐	‐	‐	Historical	1605.78	665.94	40.26	6091.02
WAL111	11693	UAM: MAMM	Female	1971	Bering Sea	Subadult	‐	‐	‐	‐	Historical	30329.86	7621.84	10412.57	9460.71
WAL126	S14–0002A	Subsistence	Male	2014	Savoonga	Adult	18	‐	‐	‐	Modern	177.73	117.02	28.52	2204.44
WAL128.1	S14–0005	Subsistence	Male	2014	Savoonga	Adult	24	‐	‐	‐	Modern	1350.89	478.61	69.05	2507.32
WAL130.1	S14–0009	Subsistence	Male	2014	Savoonga	Adult	15	‐	‐	‐	Modern	1867.40	544.63	51.73	2308.57
WAL135.1	S14–0018	Subsistence	Male	2014	Savoonga	Subadult	9	‐	‐	‐	Modern	583.23	315.14	669.50	2326.50
WAL136.1	S14–0019	Subsistence	Male	2014	Savoonga	Subadult	10	‐	‐	‐	Modern	1067.52	243.52	43.60	1984.32
WAL137.1	S14–0021	Subsistence	Male	2014	Savoonga	Adult	26	‐	‐	‐	Modern	55.38	45.05	4.64	2400.36
WAL138.1	S14–0022	Subsistence	Male	2014	Savoonga	Adult	15	‐	‐	‐	Modern	38.71	146.66	4.92	2505.16
WAL139.1	S14–0024	Subsistence	Male	2015	Savoonga	Adult	28	‐	‐	‐	Modern	5464.69	1333.68	7.34	4030.24
WAL140.1	S14–0027	Subsistence	Female	2014	Savoonga	Adult	‐	No	Calf	Y	Modern	1526.27	722.84	63.37	2483.80
WAL141.1	S14–0029	Subsistence	Male	2014	Savoonga	Adult	19	‐	‐	‐	Modern	395.55	302.16	165.50	2137.07
WAL142.1	S14–0034	Subsistence	Male	2014	Savoonga	Adult	15	‐	‐	‐	Modern	511.31	334.89	135.30	2343.49
WAL143.1	S14–0035	Subsistence	Male	2014	Savoonga	Subadult	14	‐	‐	‐	Modern	625.95	377.46	457.87	2416.88
WAL144.1	S14–0036	Subsistence	Male	2014	Savoonga	Subadult	12	‐	‐	‐	Modern	2753.11	328.88	60.23	2634.24
WAL145	S14–0038	Subsistence	Male	2014	Savoonga	Adult	18	‐	‐	‐	Modern	2572.37	279.55	38.92	2074.89
WAL146	S14–0039	Subsistence	Male	2014	Savoonga	Adult	17	‐	‐	‐	Modern	1266.98	254.52	65.24	2637.05
WAL147.1	S14–0040	Subsistence	Male	2015	Savoonga	Adult	20	‐	‐	‐	Modern	36.56	82.62	54.01	2373.95
WAL148	S14–0044	Subsistence	Male	2014	Savoonga	Adult	23	‐	‐	‐	Modern	1435.06	311.40	230.07	2343.89
WAL149.1	S14–0045	Subsistence	Female	2014	Savoonga	Adult	‐	Yes	Calf and yearling	Y	Modern	352.07	142.47	15.66	2370.49
WAL150	S14–0046	Subsistence	Male	2014	Savoonga	Adult	22	‐	‐	‐	Modern	39.78	75.67	15.44	2363.43
WAL151.1	G14–0002	Subsistence	Female	2014	Gambell	Adult	14	No	Calf and yearling	Y	Modern	283.70	234.97	349.71	2469.19
WAL153.1	G14–0011	Subsistence	Female	2014	Gambell	Adult	15	Yes	None	Y	Modern	159.05	286.52	33.71	2567.74
WAL154.1	G14–0036	Subsistence	Male	2014	Gambell	Subadult	14	‐	‐	‐	Modern	230.43	100.17	15.87	2574.39
WAL155.1	G14–0042	Subsistence	Female	2014	Gambell	Adult	9	No	Calf	Y	Modern	171.23	80.06	29.82	2483.24
WAL156.1	G14–0046	Subsistence	Female	2014	Gambell	Adult	‐	No	Calf	Y	Modern	209.46	139.07	15.08	2383.13
WAL157.1	G14–0048	Subsistence	Female	2014	Gambell	Adult	16	No	Calf	Y	Modern	112.38	86.10	14.02	2364.61
WAL189	S14–0002B	Subsistence	Male	2014	Savoonga	Adult	18	‐	‐	‐	Modern	2193.36	1188.16	202.28	2193.36
WAL234	S15–039	Subsistence	Male	2015	Savoonga	Subadult	12	‐	‐	‐	Modern	15.33	186.39	72.03	29.47
WAL235**	S15–013	Subsistence	Male	2015	Savoonga	Adult	15.5	‐	‐	‐	Modern	35.77	253.51	141.11	34.83
WAL237	S15–036	Subsistence	Male	2015	Savoonga	Adult	23.5	‐	‐	‐	Modern	6.33	132.22	49.22	13.48
WAL238	S15–030	Subsistence	Male	2015	Savoonga	Adult	15	‐	‐	‐	Modern	3.49	135.44	59.16	17.54
WAL239	S15–009	Subsistence	Male	2015	Savoonga	Adult	20.5	‐	‐	‐	Modern	18.09	299.98	153.50	83.04
WAL240	S15–022	Subsistence	Male	2015	Savoonga	Adult	19	‐	‐	‐	Modern	3.72	100.02	43.97	20.47
WAL282	G15–005	Subsistence	Female	2015	Gambell	Adult	19	No	Calf	Y	Modern	127.73	108.69	58.98	29.79
WAL283	G15–023	Subsistence	Male	2014	Gambell	Adult	20	‐	‐	‐	Modern	40.39	153.42	40.41	27.44
WAL284	G15–015	Subsistence	Male	2014	Gambell	Adult	28	‐	‐	‐	Modern	11.98	68.77	36.35	20.16
WAL59.1	11691	UAM: MAMM	Female	1972	Bering Sea	Subadult	‐	‐	‐	‐	Historical	753.68	1231.11	152.38	6479.88
WAL751	129342	UAM: MAMM	Female	1983	Chukchi Sea (Russian side)	Adult	12	No	‐	‐	Historical	259.93	397.96	34.89	59.47
WAL752	WAL752	UAM: MAMM	Male	1983	Chukchi Sea (Russian side)	Subadult	7	‐	‐	‐	Historical	1682.18	674.84	75.93	46.74
WAL753	129344	UAM: MAMM	Female	1983	Chukchi Sea (Russian side)	Adult	13	No	‐	‐	Historical	405.72	543.13	63.12	42.41
WAL758	129349	UAM: MAMM	Female	1983	Chukchi Sea (Russian side)	Adult	19	No	‐	‐	Historical	1067.48	291.01	29.57	57.16
WAL759	129350	UAM: MAMM	Female	1983	Chukchi Sea (Russian side)	Adult	10	No	‐	‐	Historical	998.81	443.51	55.50	96.43
WAL761	129353	UAM: MAMM	Female	1983	Chukchi Sea (Russian side)	Adult	22	No	‐	‐	Historical	811.95	320.01	36.63	104.41
WAL762	129354	UAM: MAMM	Female	1983	Chukchi Sea (Russian side)	Adult	28	No	‐	‐	Historical	535.30	204.49	23.98	57.16
WAL764	129356	UAM: MAMM	Female	1983	Chukchi Sea (Russian side)	Adult	14	No	‐	‐	Historical	435.49	301.95	22.41	41.06
WAL765	129357	UAM: MAMM	Female	1983	Chukchi Sea (Russian side)	Adult	16	No	‐	‐	Historical	162.51	289.20	13.61	32.01
WAL767	129359	UAM: MAMM	Female	1983	Chukchi Sea (Russian side)	Adult	21	No	‐	‐	Historical	265.55	168.34	36.01	48.55
WAL768	129360	UAM: MAMM	Female	1983	Chukchi Sea (Russian side)	Adult	19	No	‐	‐	Historical	202.55	288.58	24.20	21.93
WAL769	129361	UAM: MAMM	Female	1983	Chukchi Sea (Russian side)	Adult	26	No	‐	‐	Historical	1971.63	407.22	55.77	45.56
WAL770	11689	UAM: MAMM	Female	1983	Chukchi Sea (Russian side)	Subadult	8	No	‐	‐	Historical	228.96	465.45	32.10	51.72
WAL771	129363	UAM: MAMM	Female	1983	Chukchi Sea (Russian side)	Adult	24	Yes	‐	‐	Historical	1392.46	361.05	31.18	72.15
WAL772	129364	UAM: MAMM	Female	1983	Chukchi Sea (Russian side)	Adult	15	No	‐	‐	Historical	897.14	265.96	37.11	60.25
WAL774	129366	UAM: MAMM	Female	1983	Chukchi Sea (Russian side)	Adult	16	Yes	‐	‐	Historical	1919.66	439.11	88.70	96.40
WAL775	129367	UAM: MAMM	Female	1983	Chukchi Sea (Russian side)	Subadult	1	No	‐	‐	Historical	1696.45	1554.27	145.74	47.89
WAL776	129368	UAM: MAMM	Female	1983	Chukchi Sea (Russian side)	Adult	15	Yes	‐	‐	Historical	3414.99	541.95	20.44	167.39
WAL779	G16–003	Subsistence	Female	2016	Gambell	Adult	11	No	None	N	Modern	260.97	158.89	115.45	19.00
WAL780	G16–005	Subsistence	Female	2016	Gambell	Adult	19	No	None	N	Modern	20.68	67.20	38.16	20.68
WAL781	G16–006	Subsistence	Female	2016	Gambell	Adult	12	No	None	N	Modern	162.64	211.21	16.27	18.23
WAL782	G16–013	Subsistence	Female	2016	Gambell	Adult	13	No	Calf and yearling	N	Modern	19.16	62.49	6.43	19.16
WAL783	G16–014	Subsistence	Female	2016	Gambell	Adult	16	Yes	‐	Y	Modern	134.07	261.20	67.40	210.22
WAL785	G16–021	Subsistence	Female	2016	Gambell	Subadult	‐	No	‐	None	Modern	18.36	178.61	83.91	18.36
WAL786	G16–023	Subsistence	Female	2016	Gambell	Adult	12	Yes	‐	Y	Modern	101.16	73.20	26.19	18.09
WAL788	G16–034	Subsistence	Female	2016	Gambell	Adult	14	No	Yearling	Y	Modern	19.12	144.08	11.86	19.12
WAL789	G16–039	Subsistence	Female	2016	Gambell	Adult	8	No	None	N	Modern	541.12	307.48	153.46	17.41
WAL791	S16–002	Subsistence	Male	2016	Savoonga	Adult	18	‐	‐	‐	Modern	18.43	86.68	19.31	211.21
WAL792	S16–003	Subsistence	Female	2016	Savoonga	Adult	‐	No	Calf	Y	Modern	19.97	186.17	79.73	19.97
WAL794	S16–010	Subsistence	Male	2016	Savoonga	Adult	19	‐	‐	‐	Modern	18.75	33.97	9.05	100.01
WAL797	S16–016	Subsistence	Male	2016	Savoonga	Subadult	14	‐	‐	‐	Modern	18.04	34.94	6.35	12.43
WAL798	S16–020	Subsistence	Male	2016	Savoonga	Subadult	9	‐	‐	‐	Modern	482.50	246.88	196.99	20.94
WAL799	S16–024	Subsistence	Male	2016	Savoonga	Adult	16	‐	‐	‐	Modern	53.37	38.35	3.99	66.61
WAL802	S16–031	Subsistence	Male	2016	Savoonga	Subadult	7	‐	‐	‐	Modern	210.52	87.65	42.66	23.22
WAL804	S16–034	Subsistence	Male	2016	Savoonga	Adult	16	‐	‐	‐	Modern	20.46	40.36	8.42	23.80
WAL806	S16–037	Subsistence	Male	2016	Savoonga	Adult	20	‐	‐	‐	Modern	51.31	55.16	8.99	24.68
WAL808	S16–039	Subsistence	Male	2016	Savoonga	Adult	16	‐	‐	‐	Modern	17.44	101.62	28.42	105.77
WAL809.1	130619	UAM: MAMM	Female	1987	South Chukchi Sea	Adult	24	No	‐	‐	Historical	236.90	134.62	20.13	45.23
WAL810.1	130620	UAM: MAMM	Female	1987	South Chukchi Sea	Adult	33	No	‐	‐	Historical	128.07	206.16	15.97	45.21
WAL813.1	130621	UAM: MAMM	Female	1987	West Chukchi Sea	Adult	26	No	‐	‐	Historical	274.80	240.63	32.50	48.09
WAL814.1	130624	UAM: MAMM	Female	1987	West Chukchi Sea	Adult	21	No	‐	‐	Historical	321.15	127.90	35.21	58.13
WAL815	130625	UAM: MAMM	Female	1966	Gambell	Adult	11	Yes	Yearling	‐	Historical	360.20	458.18	27.72	62.59
WAL816.1	WAL816.1	UAM: MAMM	Male	1965	Gambell	Subadult	3	‐	‐	‐	Historical	1021.69	379.83	34.17	47.08
WAL817	WAL817	UAM: MAMM	Male	1952	Gambell	Adult	17.5	‐	‐	‐	Historical	2854.69	520.20	239.28	60.31

## APPENDIX 3

### 3.3

List of all samples collected, and tissues analyzed for steroid hormone analysis with relevant provenience and steroid hormone data. “‐” indicates no data. Samples came from 2014–2015 Native harvests on St. Lawrence Island, AK. Age class and sex determined from hunter observations. Estimated ages were based on counting cementum growth layers in the walrus teeth. Tissue labels for hormone concentrations (i.e., last four columns) are abbreviated as follows: bone (B), blubber (Bl), and serum (S). Hormones are abbreviated as follows: cortisol (C), estradiol (E), progesterone (P), and testosterone (T). All units are in ng/g of tissue or ng/mL for serum

**Table:**

Catalog name	Project number	Tissues analyzed	Sex	Reproductive info	Date collected [D‐M‐Y]	Location	Age class	Estimated age [years]	SP [ng/mL]	ST [ng/mL]	SC [ng/mL]	SE [ng/mL]	BlP [ng/g]	BlT [ng/g]	BlC [ng/g]	BlE [ng/g]	BP [ng/g]	BT [ng/g]	BC [ng/g]	BE [ng/g]
S14–0002A	WAL126	Bone, serum	Male		16‐May‐14	Savoonga	Adult	18	1.59	10.68	10.68	85.25	‐	‐	‐	‐	8.59	5.66	1.38	106.58
S14–0005	WAL128.1	Bone, blubber	Male	‐	22‐May‐14	Savoonga	Subadult	24	‐	‐	‐	‐	6.44	3.82	10.74	137.98	65.31	23.14	3.34	121.22
S14–0007	WAL129.1	Bone, blubber, serum	Male	‐	2014	Savoonga	Unknown	18	9.21	5.20	23.80	94.33	6.87	1.81	5.09	151.69	25.75	17.18	3.61	117.06
S14–0011	WAL132.1	Bone, blubber, serum	Female	None	22‐May‐14	Savoonga	Unknown	‐	5.24	10.61	23.35	89.53	21.23	9.68	7.97	112.96	21.31	56.21	2.85	100.57
S14–0014	WAL133.1	Bone, blubber	Male	‐	5‐May‐14	Savoonga	Unknown	‐	‐	‐	‐	‐	9.89	20.95	8.39	171.88	10.89	12.37	8.20	107.96
S14–0018	WAL135.1	Bone, blubber	Male	‐	22‐May‐14	Savoonga	Adult	9	‐	‐	‐	‐	4.04	12.52	13.17	126.28	28.20	15.24	32.37	112.48
S14–0021	WAL137.1	Bone, blubber	Male	‐	22‐May‐14	Savoonga	Unknown	26	‐	‐	‐	‐	3.92	11.18	3.69	129.48	2.68	2.18	0.22	116.05
S14–0022	WAL138.1	Bone, blubber, serum	Male	‐	2014	Savoonga	Subadult	15	2.32	14.79	16.89	160.44	5.57	14.20	5.74	116.33	1.87	7.09	0.24	121.12
S14–0024	WAL139.1	Bone, blubber	Male	‐	4‐May‐14	Savoonga	Subadult	28	‐	‐	‐	‐	3.27	24.62	4.47	109.51	264.20	64.48	0.36	194.85
S14–0029	WAL141.1	Bone, blubber, serum	Male	‐	24‐May‐14	Savoonga	Adult	19	3.80	14.03	19.69	89.64	5.06	21.59	5.98	106.93	19.12	14.61	8.00	103.32
S14–0034	WAL142.1	Bone, blubber, serum	Male	‐	22‐May‐14	Savoonga	Adult	15	4.80	9.15	14.84	94.05	2.95	12.60	2.34	111.35	24.72	16.19	6.54	113.30
S14–0035	WAL143.1	Bone, blubber	Male	‐	11‐May‐14	Savoonga	Adult	14	‐	‐	‐	‐	5.39	12.03	4.29	110.98	30.26	18.25	22.14	116.85
S14–0036	WAL144.1	Bone, blubber, serum	Male	‐	22‐May‐14	Savoonga	Adult	12	3.43	7.01	23.91	86.53	5.48	8.12	5.30	125.22	133.10	15.90	2.91	127.36
S14–0038	WAL145	Bone, blubber	Male	‐	4‐May‐14	Savoonga	Adult	18	‐	‐	‐	‐	3.51	5.27	4.00	112.36	124.37	13.52	1.88	100.31
S14–0039	WAL146	Bone, blubber	Male	‐	4‐May‐14	Savoonga	Adult	17	‐	‐	‐	‐	15.71	7.66	5.96	132.76	61.25	12.31	3.15	127.49
S14–0040	WAL147.1	Bone, blubber, serum	Male	‐	4‐May‐14	Savoonga	Adult	20	0.93	7.09	27.80	96.81	7.43	8.80	5.29	118.63	1.77	3.99	2.61	114.77
S14–0044	WAL148	Bone, blubber	Male	‐	4‐May‐14	Savoonga	Adult	23	‐	‐	‐	‐	9.14	6.50	10.12	120.34	38.93	15.05	11.12	113.32
S14–0045	WAL149.1	Bone, blubber, serum	Female	Calf, Yearling, and Lactating	4‐May‐14	Savoonga	Adult	16	3.24	5.96	66.39	95.75	68.35	6.24	8.55	118.75	17.02	6.89	0.76	114.61
G14–0002	WAL151.1	Bone, serum	Female	Calf and Lactating	25‐May‐14	Gambell	Adult	14	8.00	9.52	25.53	99.75	‐	‐	‐	‐	13.72	11.36	16.91	119.38
G14–0005	WAL152.1	Bone, blubber, serum	Male	‐	4‐May‐14	Gambell	Adult	‐	5.00	9.09	12.75	83.57	8.41	5.33	3.59	116.77	26.01	19.16	118.84	100.84
G14–0011	WAL153.1	Bone, blubber, serum	Female	Pregnant	17‐May‐14	Gambell	Adult	15	4.40	4.64	33.08	82.91	141.98	16.79	7.48	127.60	7.69	13.85	1.63	124.14
G14–0036	WAL154.1	Bone, blubber	Male	‐	4‐May‐14	Gambell	Adult	14	‐	‐	‐	‐	3.01	19.84	6.35	121.96	11.14	4.84	0.77	124.46
G14–0046	WAL156.1	Bone, blubber, serum	Female	Calf and Lactating	4‐May‐14	Gambell	Adult	‐	4.40	7.68	80.37	91.49	85.07	7.57	4.35	113.84	10.13	6.72	0.73	115.22
S15–027	WAL233	Bone, blubber	Male	‐	2015	Savoonga	Unknown	‐	‐	‐	‐	‐	1.07	1.77	1.13	1.97	1.19	19.00	13.60	1.44
S15–039	WAL234	Bone, blubber, serum	Male	‐	10‐May‐15	Savoonga	Adult	11–13	8.00	7.18	28.85	1.33	1.06	4.44	0.66	2.35	0.74	9.01	3.48	1.42
S15–013	WAL235**	Bone, blubber, serum	Male	‐	11‐May‐15	Savoonga	Adult	15–16	3.00	5.38	15.01	0.93	5.61	2.26	2.07	1.05	1.73	12.26	6.82	1.68
S15–036	WAL237	Bone, blubber, serum	Male	‐	11‐May‐15	Savoonga	Adult	23–24	1.55	7.59	19.70	1.55	0.99	1.73	2.55	0.99	0.31	6.39	2.38	0.65
S15–030	WAL238	Bone, blubber, serum	Male	‐	10‐May‐15	Savoonga	Adult	15	3.95	4.96	16.33	4.60	0.90	1.68	0.82	0.90	0.17	6.55	2.86	0.85
S15–009	WAL239	Bone, blubber, serum	Male	‐	7‐May‐15	Savoonga	Adult	20–21	3.23	7.64	12.58	2.28	0.99	2.04	1.58	0.99	0.87	14.50	7.42	4.01
S15–022	WAL240	Bone, blubber, serum	Male	‐	9‐May‐15	Savoonga	Adult	19	0.92	5.64	29.92	0.84	0.98	3.37	0.72	0.98	0.18	4.84	2.13	0.99
S15–037	WAL241	Bone, blubber	Male	‐	9‐May‐15	Savoonga	Adult	‐	‐	‐	‐	‐	1.04	4.20	1.03	1.04	0.86	6.55	3.68	0.97
G15–005	WAL282	Bone, blubber, serum	Female	Yearling and Lactating	7‐May‐15	Gambell	Adult	19–20	18.04	2.87	67.72	1.34	110.87	1.02	2.42	0.97	6.18	5.25	2.85	1.44
G15–015	WAL283	Bone, blubber, serum	Male	‐	15‐May‐15	Gambell	Adult	27–29	14.95	8.15	33.39	2.37	0.97	0.54	1.51	0.97	0.58	3.32	1.76	0.97
G15–023	WAL284	Bone, blubber, serum	Male	‐	15‐May‐15	Gambell	Adult	20	20.46	12.37	26.66	0.77	0.89	1.65	0.83	0.89	1.95	7.42	1.95	1.33
